# Supplementary material for: Analysis of Arabidopsis non-reference accessions reveals high diversity of metabolic gene clusters and discovers new candidate cluster members
Source: Front Plant Sci. 2023 Jan 26;14:1104303. doi: 10.3389/fpls.2023.1104303 (PMC9909608; doi:10.3389/fpls.2023.1104303)
Supplement: Supplemental File 2 — Supplemental information and Supplemental Figures S1-S21. [file DataSheet_2.pdf]

Supplemental Figures

Figure S1. IGV screens of genomic regions covering Arabidopsis MGCs

Figure S2. Copy number analysis of genes in thalianol (A), marneral (B) and tirucalladienol (C) gene clusters

Figure S3. Copy number analysis of genes in arabidiol/baruol gene cluster

Figure S4. Evidence supporting manual correction of genotype assignments in individual genes and accessions

Figure S5. WGS data-based evidence for a new type of deletion in the thalianol gene cluster spanning *CYP705A5*, *CYP708A2* and *THAS1*

Figure S6. Duplication of acyltransferase gene in Mitterberg-2-185

Figure S7. Partial deletion of *CYP705A12* in Mir-0

Figure S8. Differences between the countries in read coverage and mapping indicate that structural variants in tirucalladienol cluster genes are of local origin

Figure S9. Alternative *CYP716A2* gene models

Figure S10. Variation in WGS data coverage and mapping in the region spanning *CYP705A2*, *CYP705A3* and *BARS1* genes

Figure S11. *CYP705A2* duplication detected by RD assay correlates with the occurrence of pseudo-heterozygous SNPs in *CYP705A2* and *BARS1* loci

Figure S12. Multiple sequence alignment of *BARS1* genomic sequences reveals a common lack of the largest intron

Figure S13. Comparison of baruol synthase 1 protein NP_001329547.1 with proteins encoded by *BARS2* genes in Cvi-0, Eri-1 and Ler-0

Figure S14. Heterozygous SNPs in Cvi-0 co-localize with sequence differences between BARS1and its duplicate

Figure S15. Sequence comparison of *CYP705A2* and its duplicate *CYP705A2a*

Figure S16. PCR verification of group assignments based on the presence/absence of *CYP705A2*, *BARS1*, *CYP705A2a* and *BARS2* genes

Figure S17. Spread of PP-AA and PP-PP variants of arabidiol/baruol gene cluster in Arabidopsis population

Figure S18. Latitudes of origin among accessions with and without *CYP705A2a-BARS2* genes divided by country

Figure S19. Variability of the arabidiol/baruol gene cluster organization better explains latitudinal distribution of Arabidopsis accessions compared to variability of the thalianol gene cluster

Figure S20. Structural variation of the thalianol gene cluster has little impact on gene expression and root growth phenotypic variation

Figure S21. Differences in expression of arabidiol/baruol gene cluster among the accessions

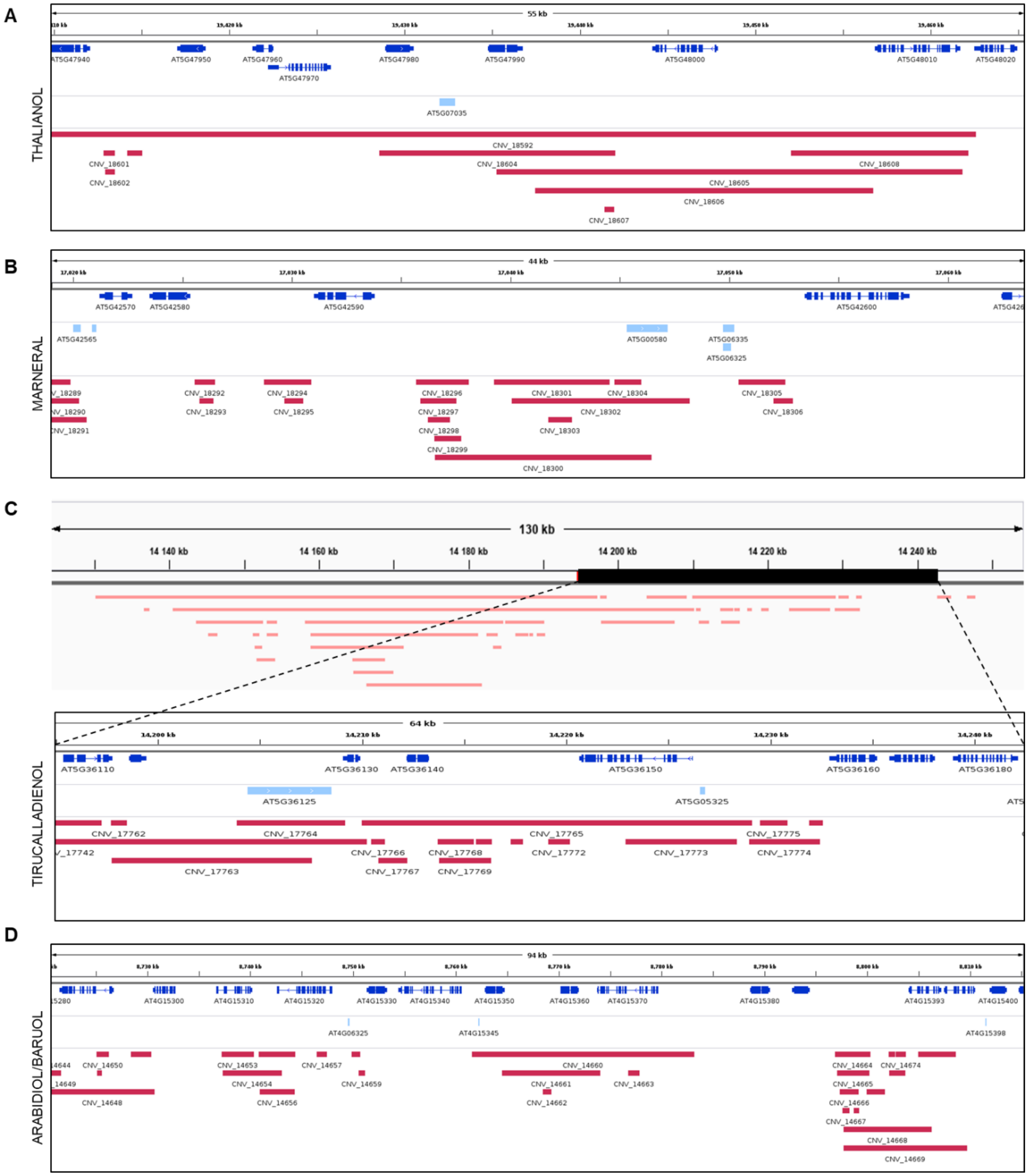

Figure S1. IGV screens of genomic regions covering Arabidopsis MGCs. A) Thalianol gene cluster; B) Marneral gene cluster; C) Tirucalladienol gene cluster; D) Arabidiol/baruol gene cluster. Dark blue – protein-coding genes. Araport 11 genomic loci are denoted. Light blue – noncoding genes. Red – CNVs extracted from AthCNV atlas (http://athcnv.ibch.poznan.pl/).

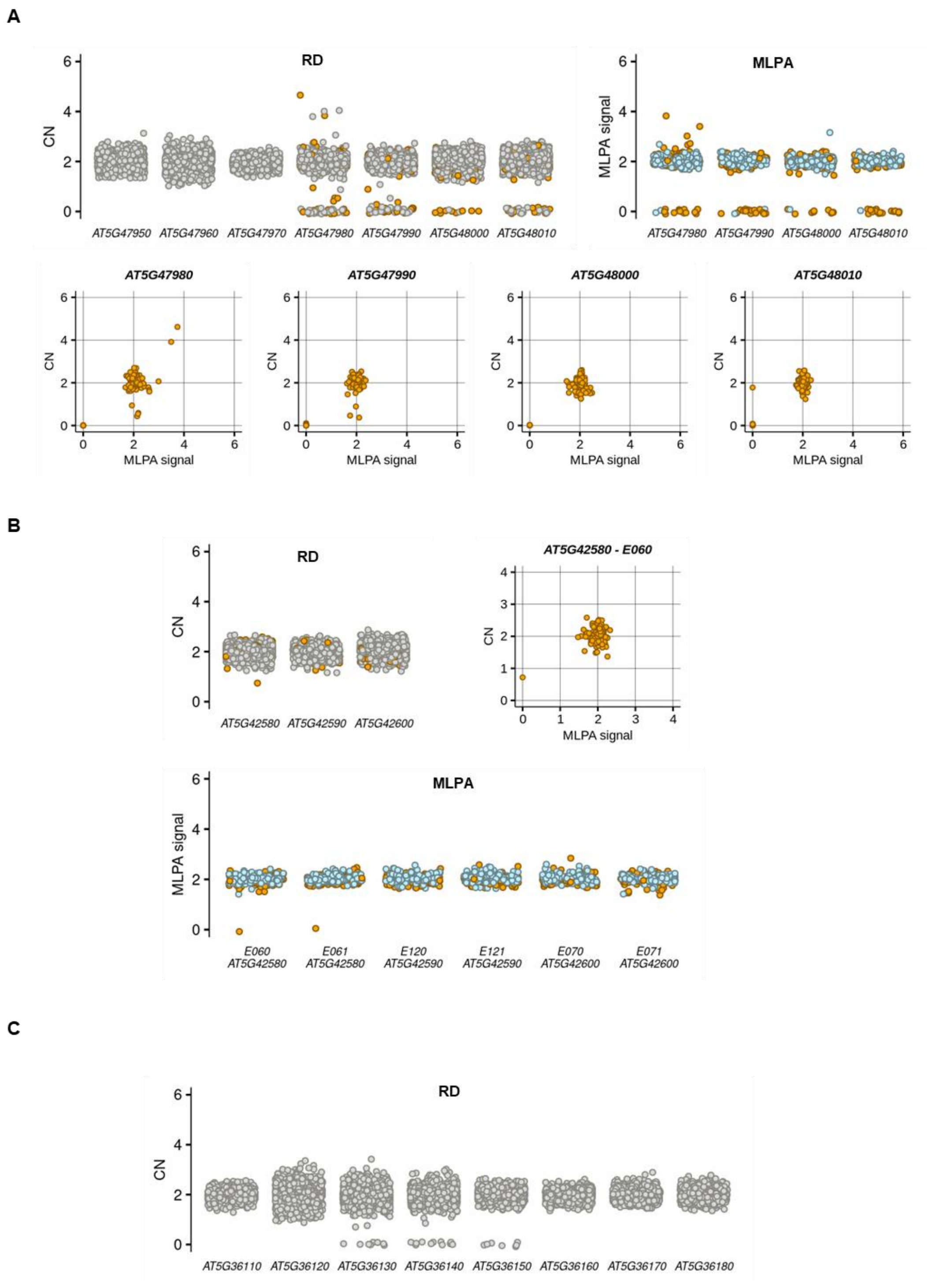

Figure S2. Copy number analysis of genes in thalianol (A), marneral (B) and tirucalladienol (C) gene clusters. Dots indicate individual accessions. Colours indicate a method of analysis: grey – RD only, blue – MLPA only, orange – both. The sets of analysed accessions differ between the methods (1,056 for RD; 232 for MLPA) but are identical for each gene.

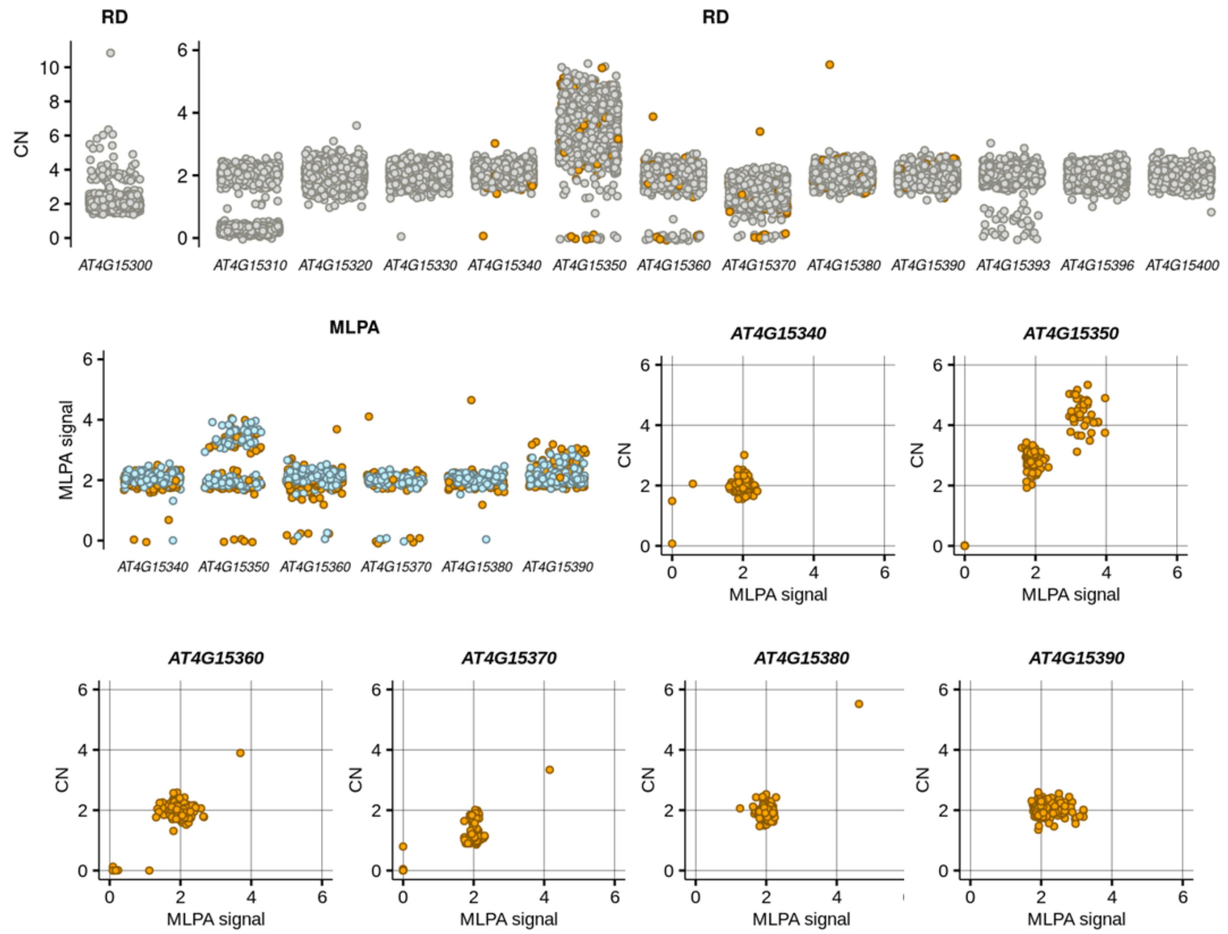

Figure S3. Copy number analysis of genes in arabidiol/baruol gene cluster. Dots indicate individual accessions. Colours indicate a method of analysis: grey – RD only, blue – MLPA only, orange – both. The sets of analysed accessions differ between the methods (1,056 for RD; 232 for MLPA) but are identical for each gene.

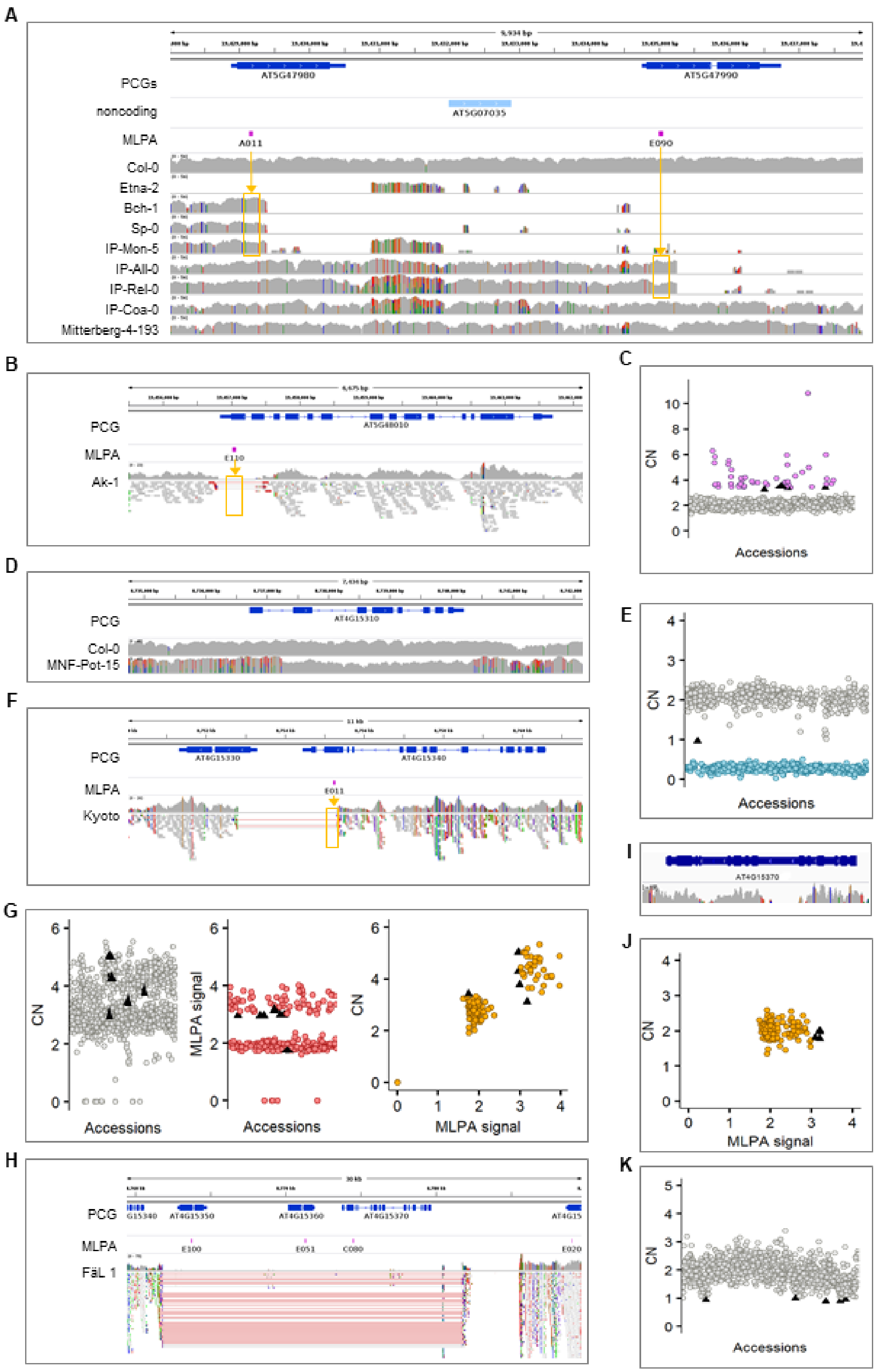

Figure S4. Evidence supporting manual correction of genotype assignments in individual genes and accessions. Details are presented in Supplemental Table S7.

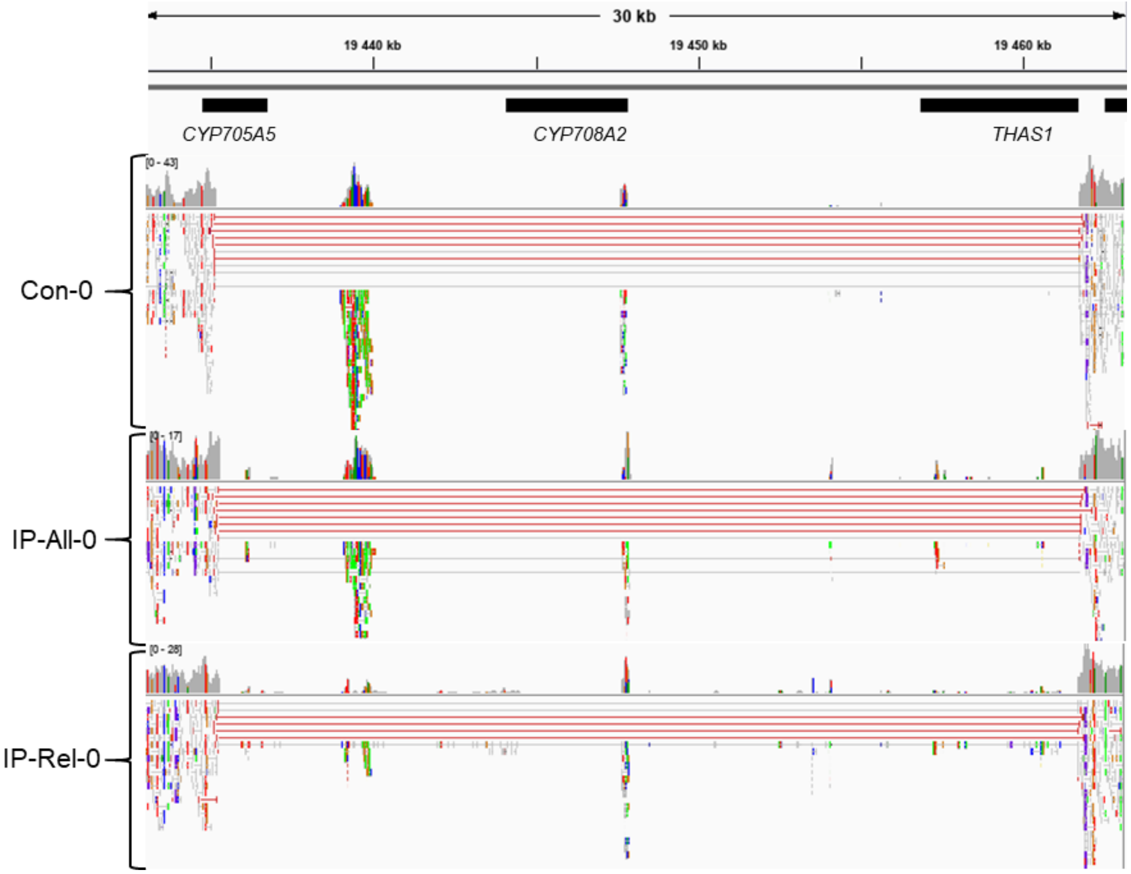

Figure S5. WGS data-based evidence for a new type of deletion in the thalianol gene cluster spanning *CYP705A5*, *CYP708A2* and *THAS1*.

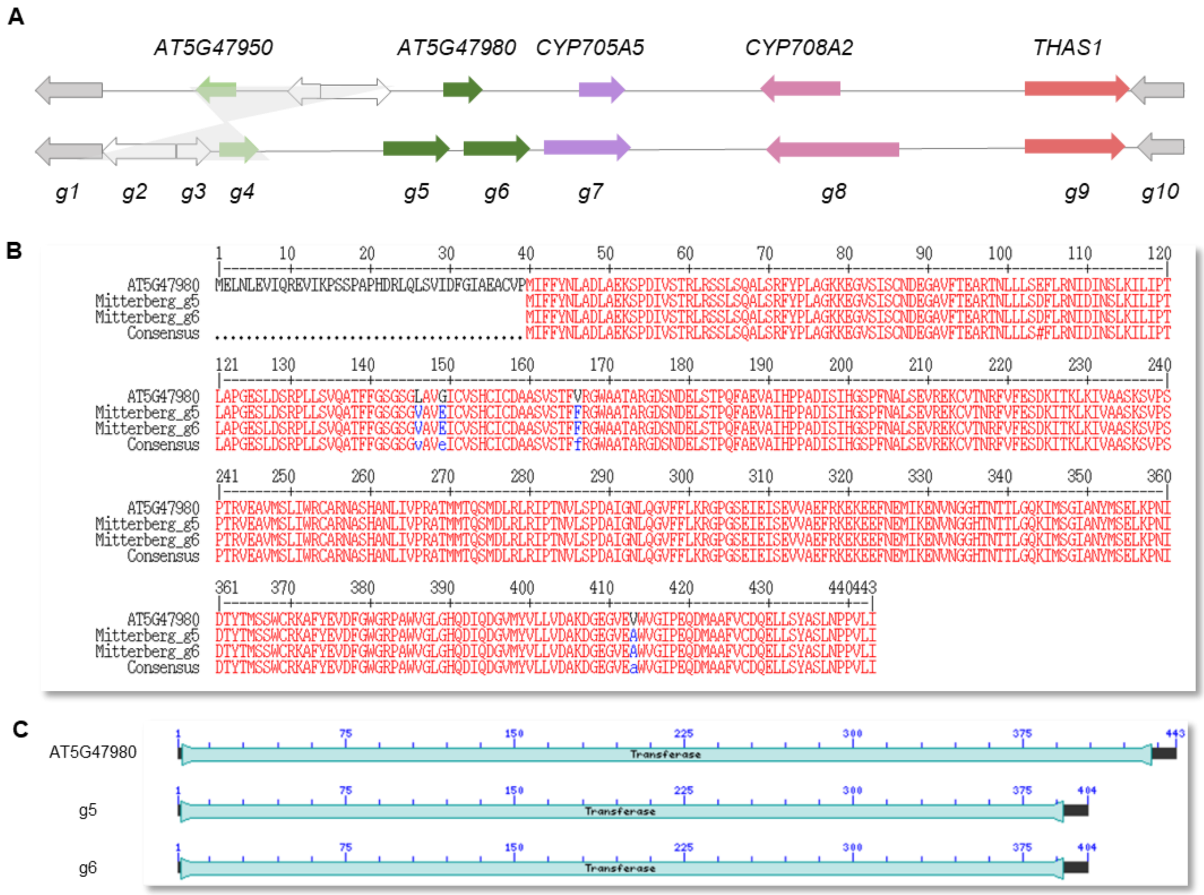

Figure S6. Duplication of acyltransferase gene in Mitterberg-2-185. A) Thalianol gene cluster organization in Col-0 (upper) and Mitterberg-2-185 *de novo* assembly (lower). Corresponding genes have the same colors. The region of inversion is marked in grey. B) Sequence alignment of reference AT5G47980 protein with predicted proteins from Mitterberg-2-185. C) Conserved domain prediction. Transferase – transferase domain (pfam02458).

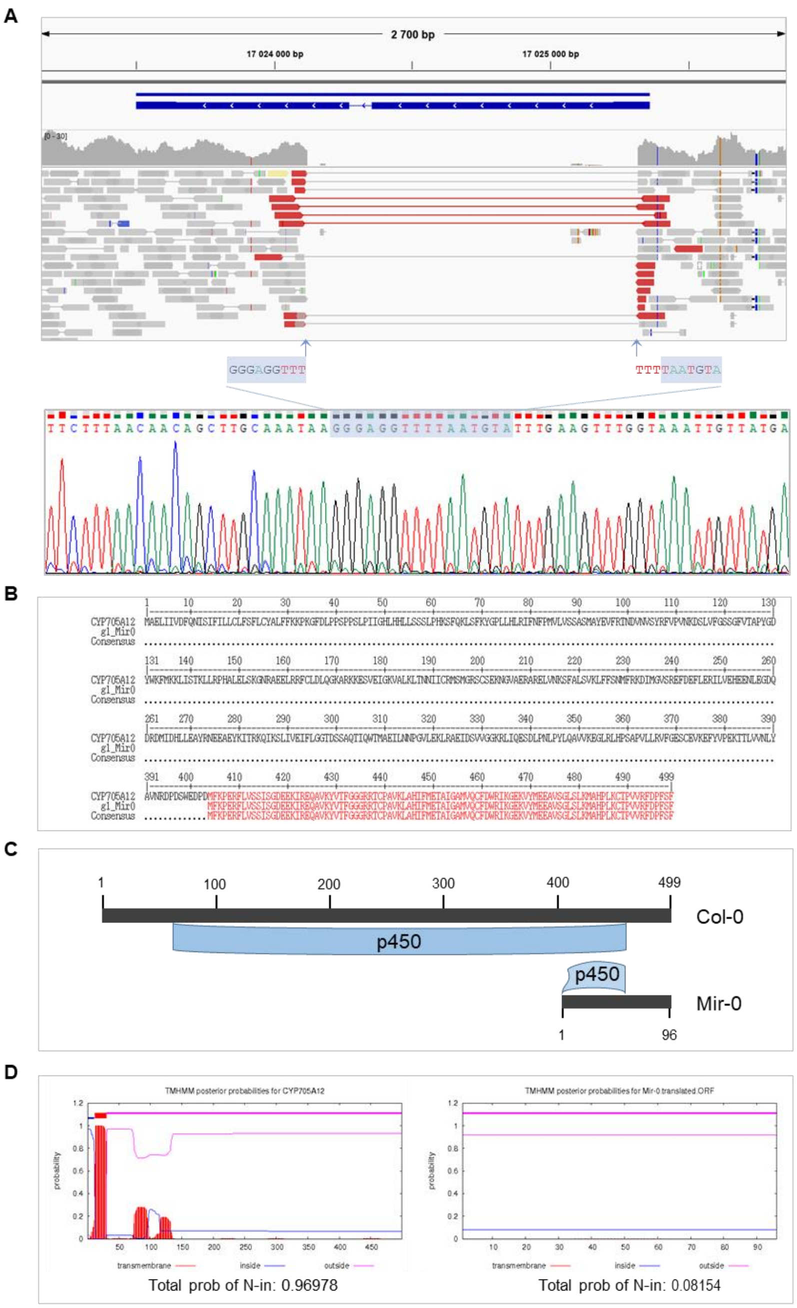

Figure S7. Partial deletion of *CYP705A12* in Mir-0. A) WGS-based and Sanger sequencing-based data concordantly indicate there is a 1,202-bp deletion in *CYP705A12* gene sequence in Mir-0. The reference *CYP705A12* model is presented in blue. B) Predicted truncated protein in Mir-0. Multiple protein alignment made with Multalin. C) Full (reference) and partial (Mir-0) cytochrome P450 superfamily domain present in the respective proteins. D) TMHMM server prediction probabilities for CYP705A12 (left) and its truncated homolog in Mir-0 (right).

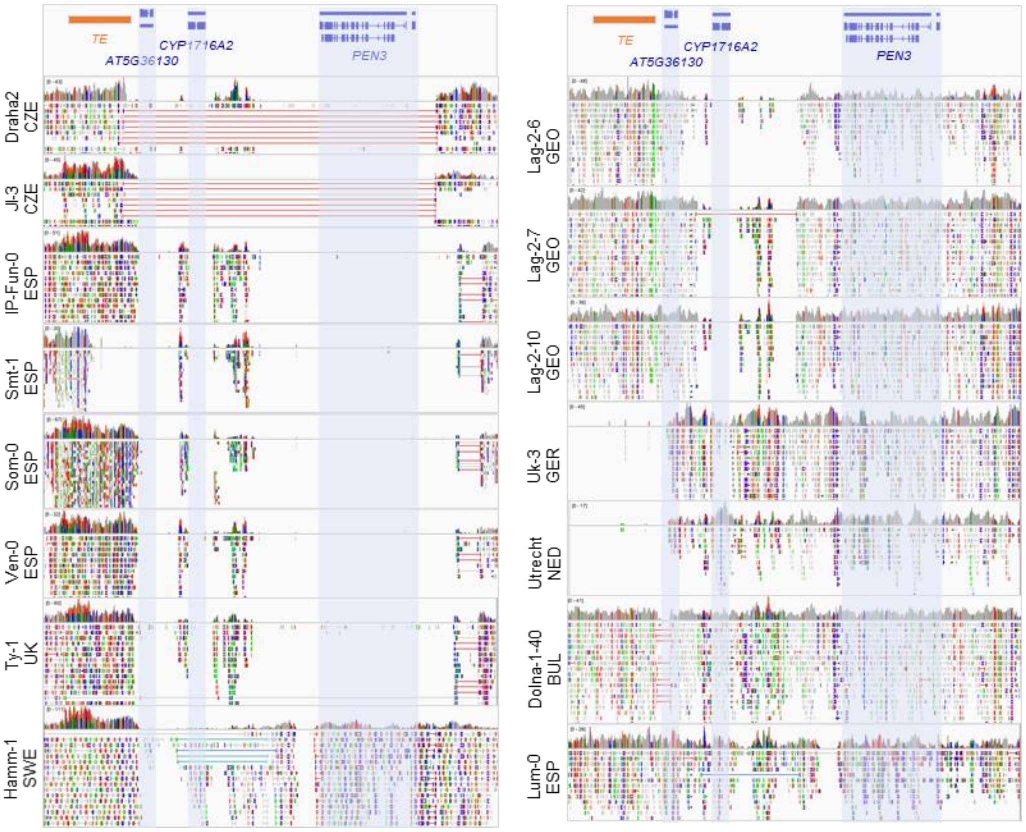

Figure S8. Differences between the countries in read coverage and mapping indicate that structural variants in tirucalladienol cluster genes are of local origin. The picture presents data for all 15 accessions with detected copy number changes in tirucalladienol cluster genes.

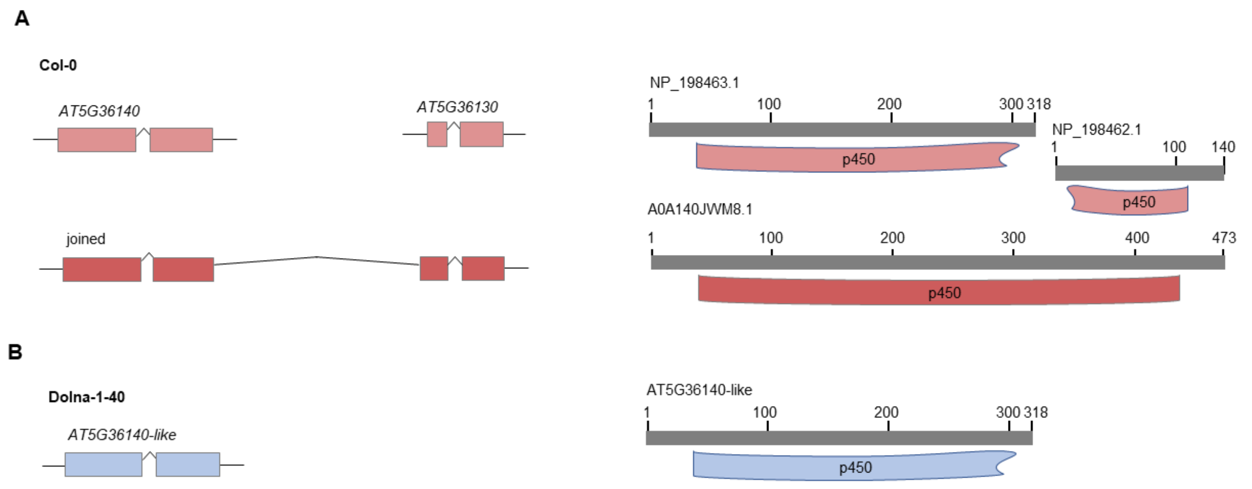

Figure S9. Alternative *CYP716A2* gene models. A) According to Araport 11 annotation, there are two separate genes, *AT5G36130* and *AT5G36140* (*CYP716A2*) in the genome. The Augustus tool predicted the same gene models and additionally an alternative joint model. The joint gene encodes a protein identical to the one predicted by Yatsumoto et al. (2016), for which full-length cDNA had been isolated. B) In Dolna-1-40 *AT5G36130* is absent, as indicated by the analysis of its *de novo* genomic assembly. The predicted ORF encodes a protein identical to *AT5G36140*, which lacks C-part of p450 superfamily domain (cl12078).

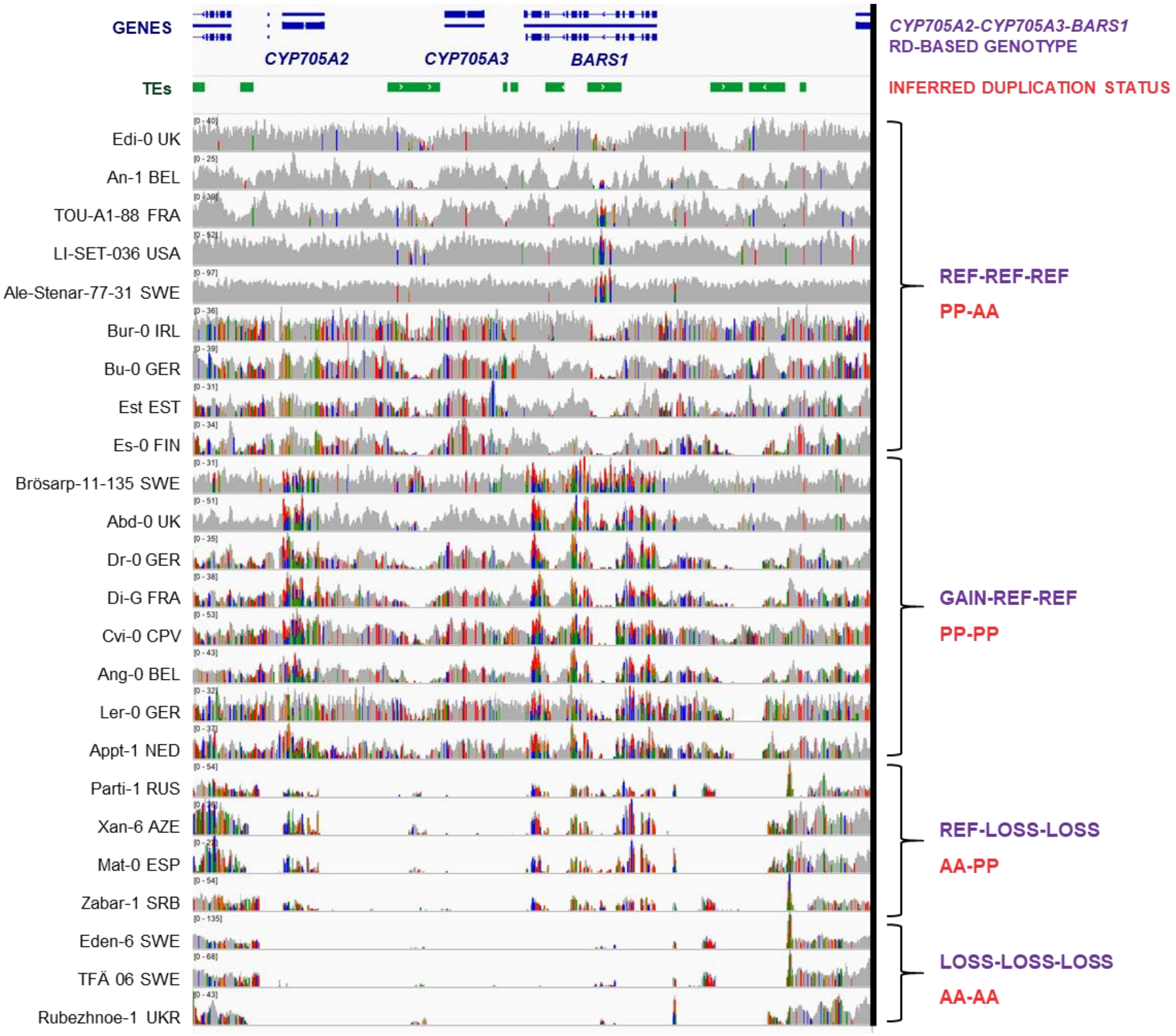

Figure S10. Variation in WGS data coverage and mapping in the region spanning *CYP705A2*, *CYP705A3* and *BARS1* genes. The presence (P) / absence (A) of *CYP705A2, BARS1* and their duplicates was inferred based on a combination of RD genotyping results and the SNP analysis at *CYP705A2* and *BARS1* loci (see Supplemental Table S11 and Methods for details).

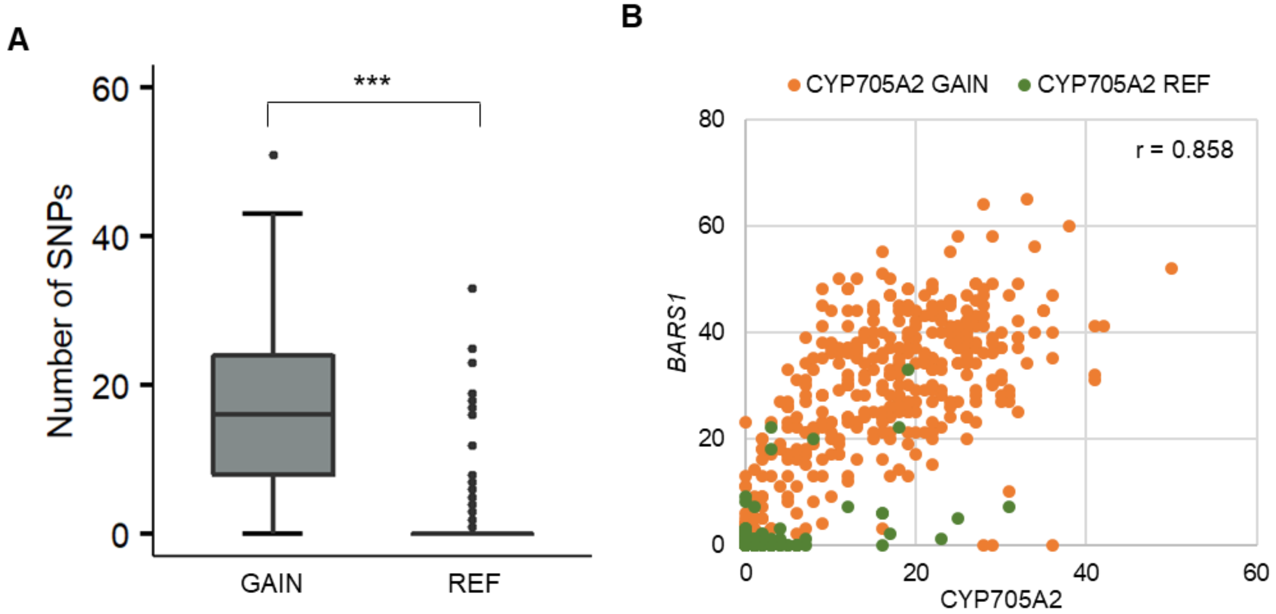

Figure S11. *CYP705A2* duplication detected by RD assay correlates with the occurrence of heterozygous SNPs at *CYP705A2* and *BARS1* loci. A) Number of heterozygous SNPs found in *CYP705A2* coding sequence in accessions with GAIN and REF genotypes. Boxplots show median (inner line) and inner quartiles (box). Whiskers extend to the highest and lowest values no greater than 1.5 times the inner quartile range. Asterisks indicate statistical significance (Wilcoxon rank sum test with continuity correction, ***p.value <0.001). B) Correlation of heterozygous SNP frequency between *CYP705A2* and *BARS1*. Colors indicate accessions with varying *CYP705A2* copy numbers. r – Pearson's correlation coefficient.

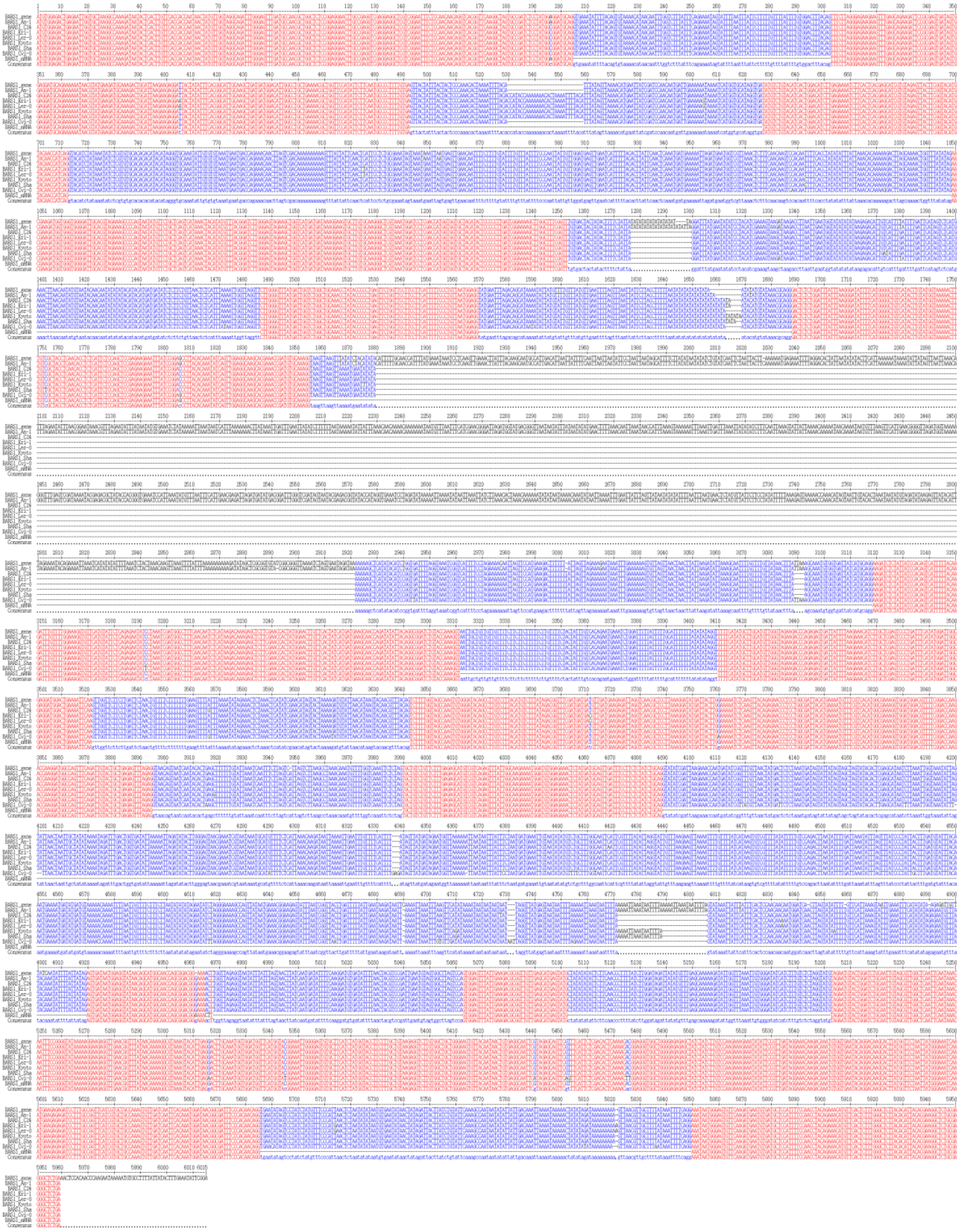

Figure S12. Multiple sequence alignment of *BARS1* genomic sequences reveals a common lack of the largest intron. Sequence order from the top: Col-0; An-1; C24; Eri-1; Ler-0; Kyoto; Sha; Cvi-0; BARS1_mRNA (to indicate exon-intron organization) and consensus.

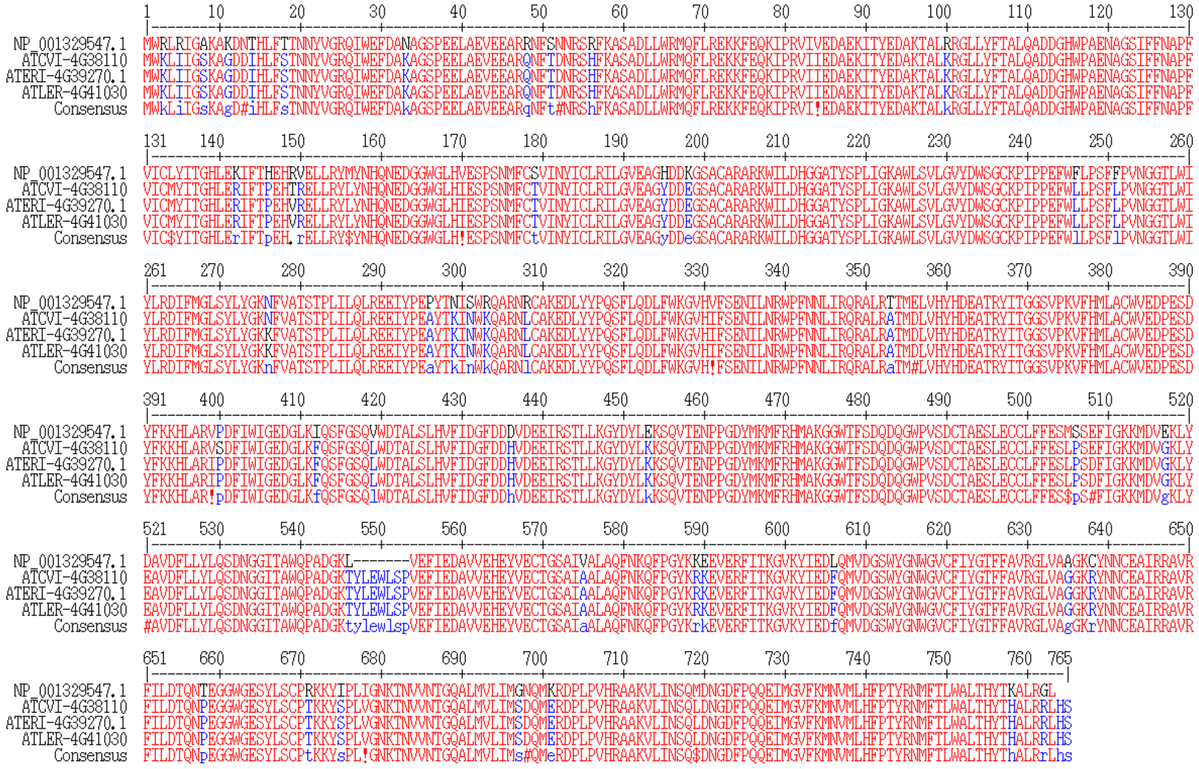

Figure S13. Comparison of baruol synthase 1 protein NP_001329547.1 with proteins encoded by *BARS2* genes in Cvi-0, Eri-1 and Ler-0. Multiple sequence alignment generated with Multalin with default parameters.

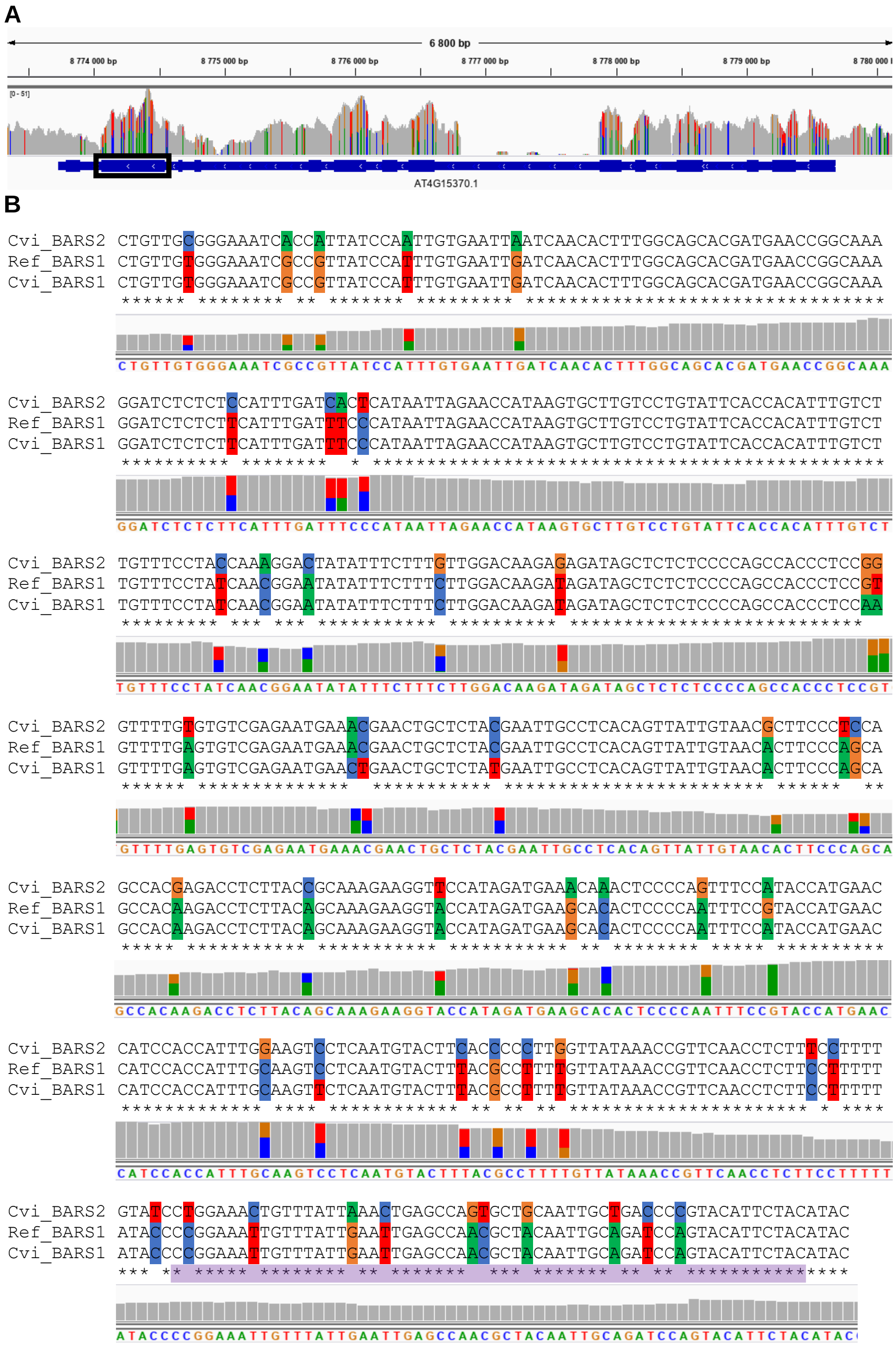

Figure S14. Heterozygous SNPs in Cvi-0 co-localize with sequence differences between BARS1and its duplicate. A) Read coverage at BARS1 locus for Cvi-0 (reads mapped to the reference genome). B) Multiple sequence alignment of the *BARS1* fragment (region chr4:8774060-8774546, marked by the black box in A) with *BARS1* and its duplicate (denoted as *BARS2*) from Cvi-0. IGV screenshots presenting Cvi-0 read coverage and the SNP positions are overlaid. The purple block indicates the position of the C080 MLPA probe.

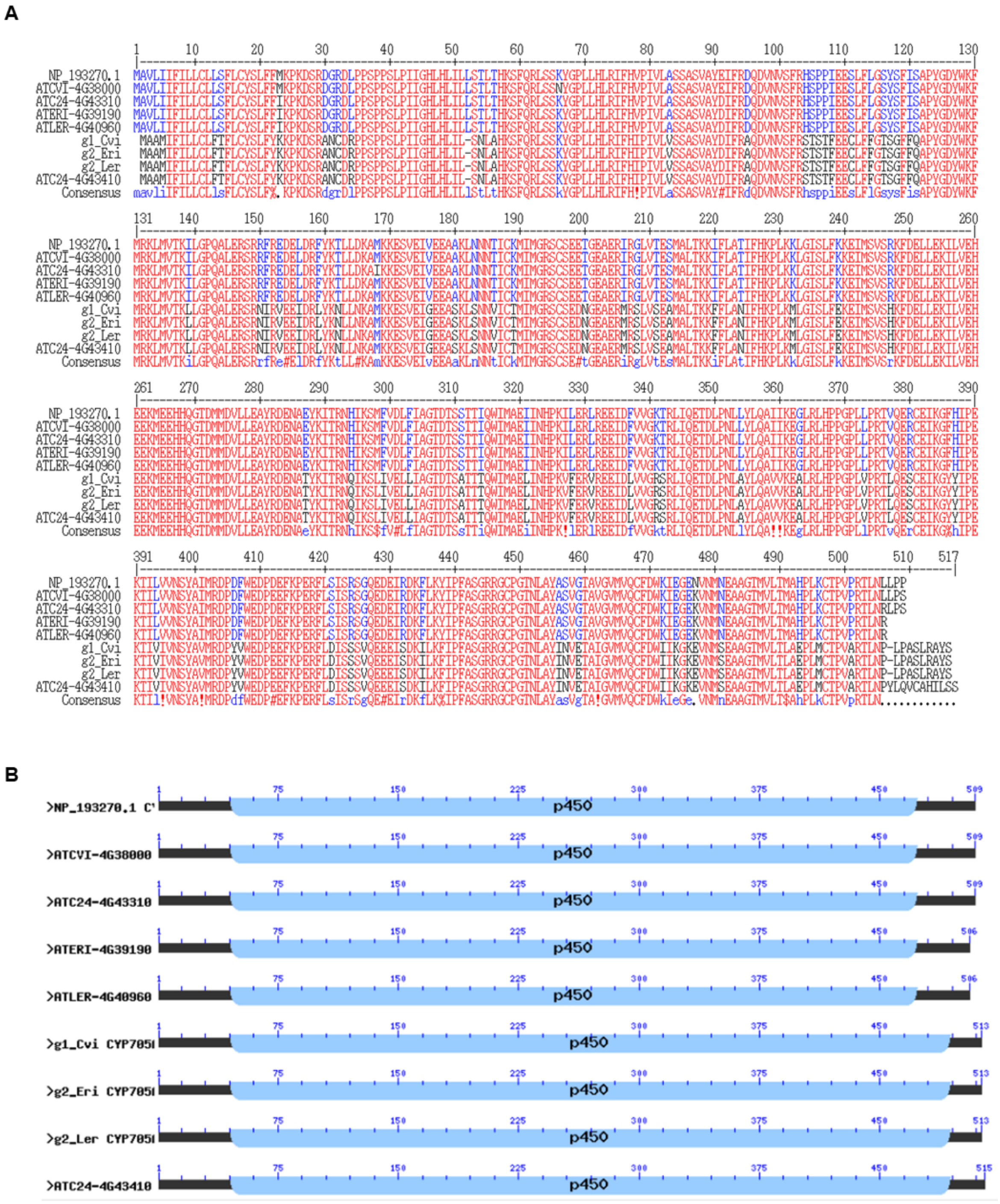

Figure S15. Sequence comparison of *CYP705A2* and its duplicate *CYP705A2a*. A) Multiple protein alignment of the proteins encoded by *CYP705A2* in Col-0, Cvi-0, C24, Eri-1 and Ler-0 (NP_193270.1, ATCVI-4G38000, ATC24-4G43310, ATERI-4G39190, ATLER-4G40960, respectively) and proteins encoded by CYP705A2 in Cvi-0, Eri-1, Ler-0 and C24 (g1_Cvi, g2_Eri, g2_Ler, ATC24-4G43410, respectively). B) Conserved protein domains in CYP705A2 and CYP705A2a sequences found by searching the Pfam database.

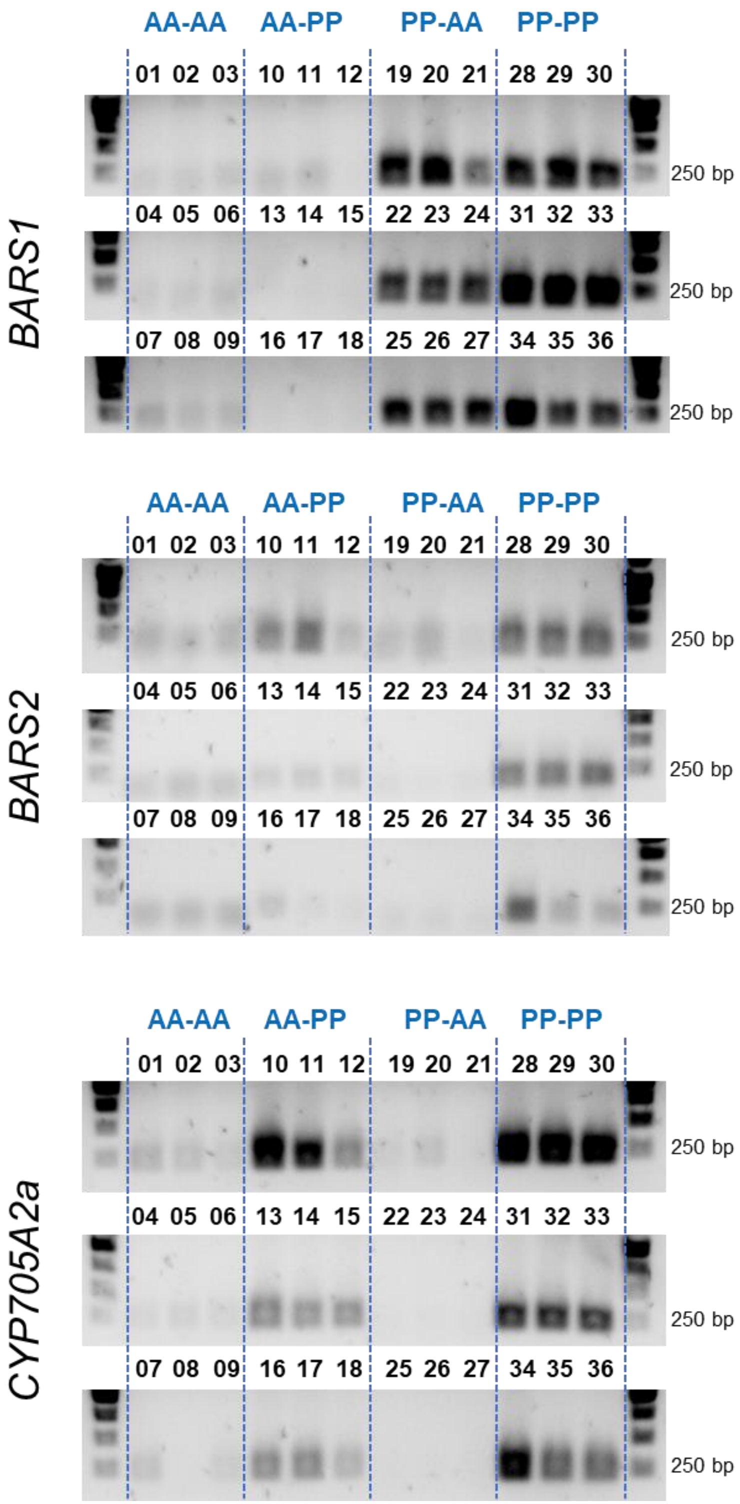

Figure S16. PCR verification of group assignments based on the presence/absence of *BARS1*, *CYP705A2a* and *BARS2* genes. Sample identities are provided in Supplemental Table S11.

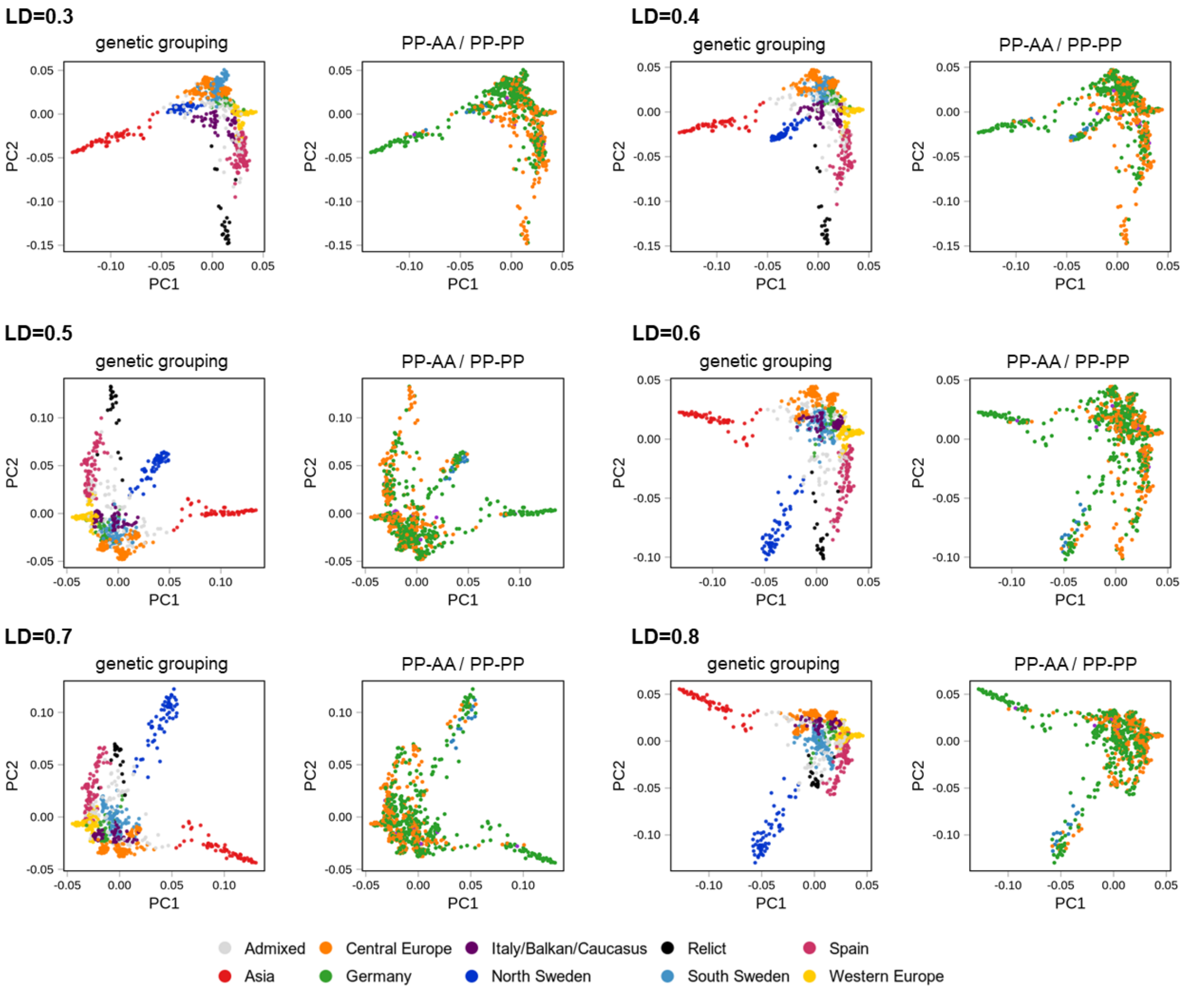

Figure S17. Spread of PP-AA and PP-PP variants of arabidiol/baruol gene cluster in Arabidopsis population. Principal component analysis (PCA) plots were generated with varying LD parameter. U.S.A. accessions were excluded from the analysis to better visualize other groups. Plots are colored according to main genetic groups (left) or *CYP705A2-BARS1* duplication status (right). Plots generated at LD=0.3 are also presented in Fig. 4 in the main text.

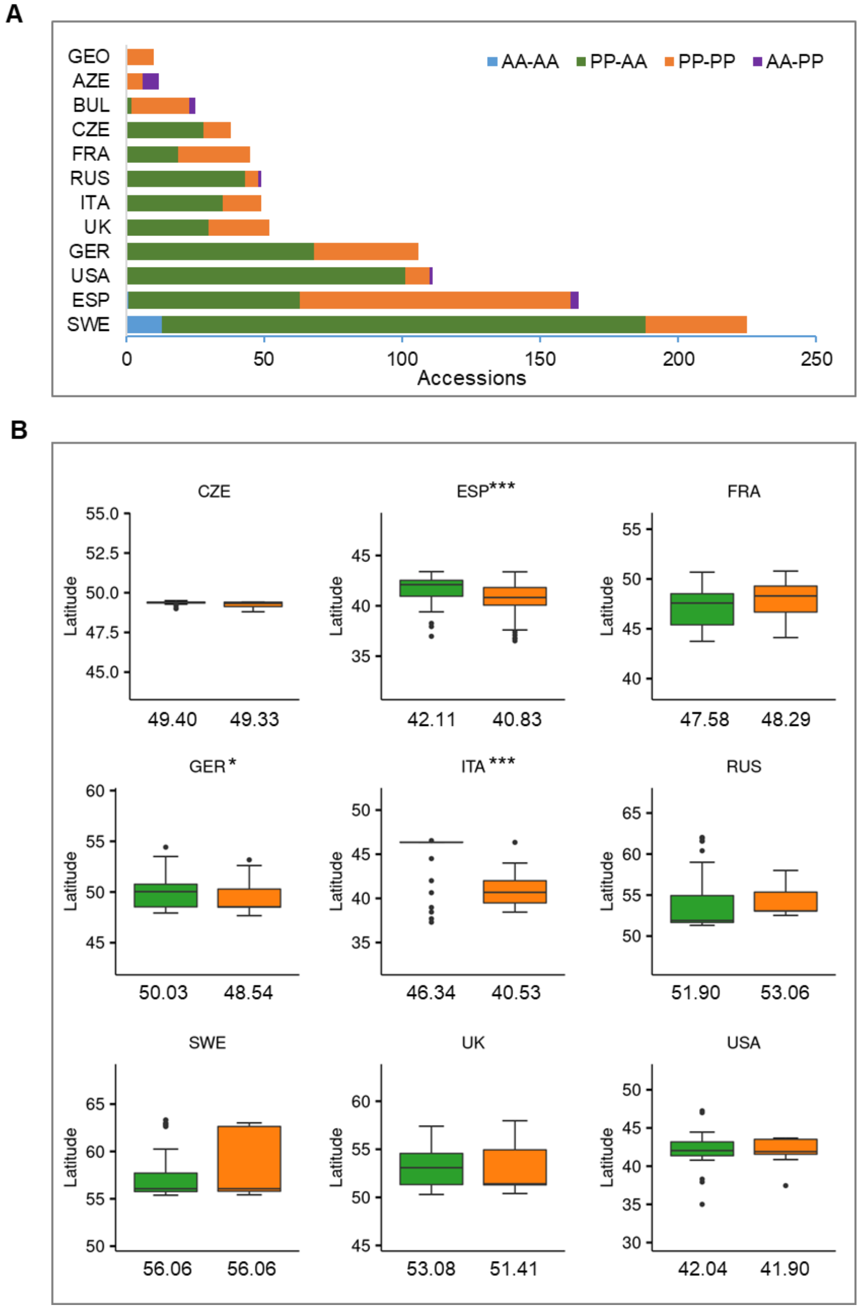

Figure S18. Latitudes of origin among accessions with and without *CYP705A2a-BARS2* genes divided by country. A) Frequency of four groups in individual countries. B) Boxplots presenting collection site latitudes of accessions from PP-AA and PP-PP groups. Only countries with ≥5 accessions within each group are presented. Median values are presented below the boxplots. Boxplots show median and inner quartiles. Whiskers extend to the highest and lowest values no greater than 1.5 times the inner quartile range. Asterisks indicate statistical significance (Wilcoxon rank sum test with continuity correction, *p.value<0.05, ***p.value <0.001).

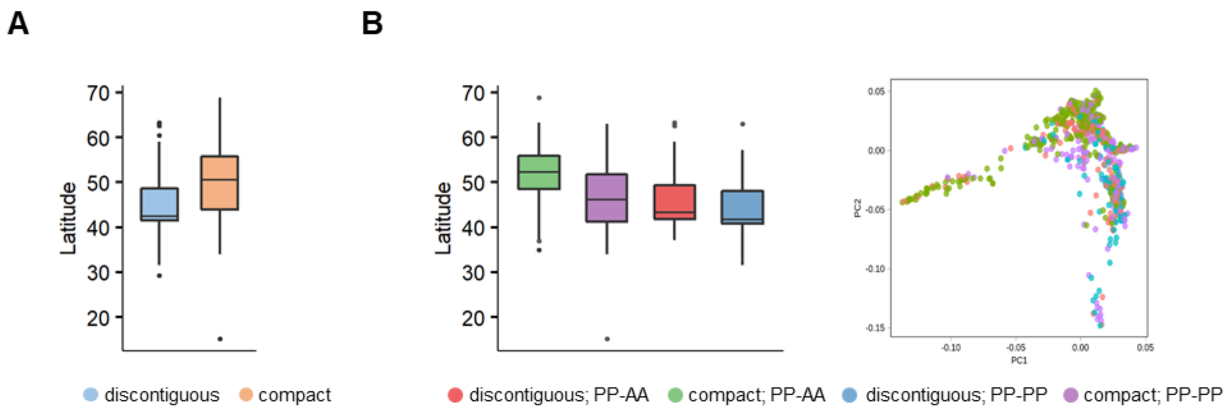

Figure S19. Variability of the arabidiol/baruol gene cluster organization better explains latitudinal distribution of Arabidopsis accessions compared to variability of the thalianol gene cluster. A) Differences in latitudinal distribution of accessions with the compact and discontiguous versions of the thalianol gene cluster. B) PCA on >200k SNPs and LD = 0.3 (left) and latitudes of origin (right) of accessions divided by both thalianol and arabidiol/baruol cluster type.

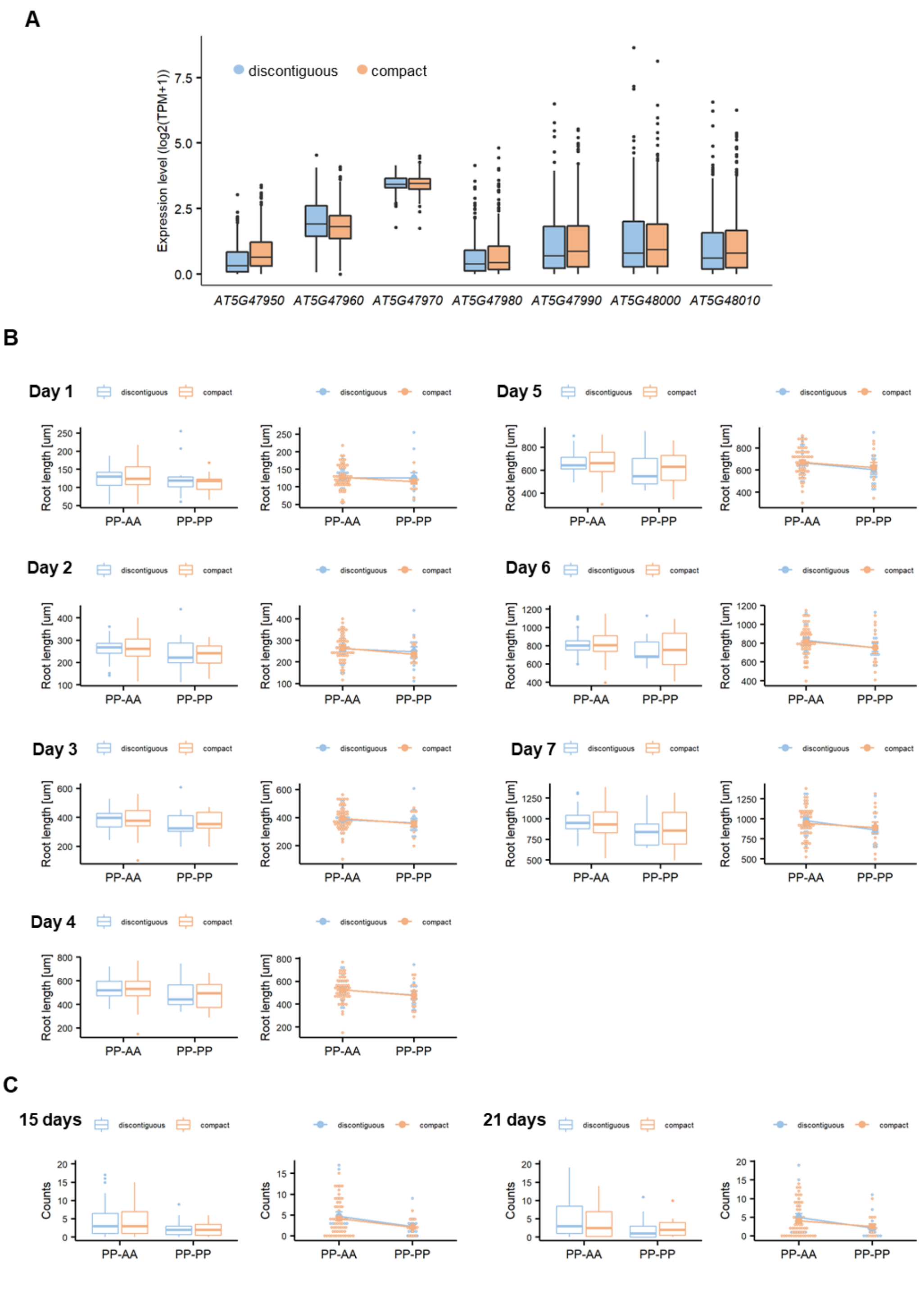

Figure S20. Structural variation of the thalianol gene cluster has little impact on gene expression and root growth phenotypic variation. A) Expression of the thalianol cluster genes in leaves in accessions with discontiguous and compact versions. B, C) Root growth phenotypes presented in the main text in Fig. 5B and 5C, respectively, divided by both thalianol and arabidiol/baruol cluster type (left), along with two-way ANOVA plot (right).

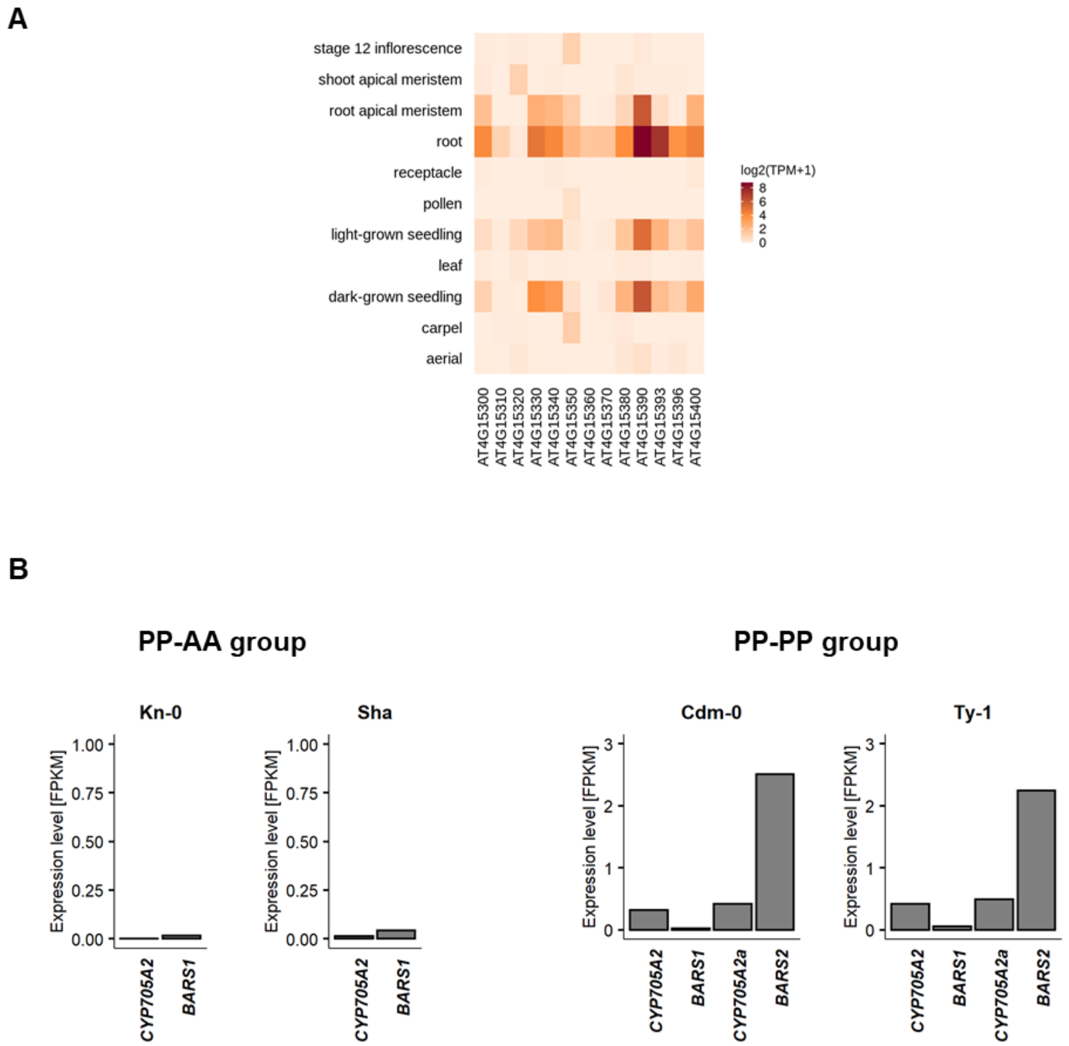

Figure S21. Differences in expression of arabidiol/baruol gene cluster among the accessions. A) Tissue-specific expression in Col-0 accession (PP-AA group). B) Expression of *CYP705A2*, *BARS1*, *CYP705A2a* and *BARS2* genes in accessions from PP-AA and PP-PP groups. For each accession, RNA-Seq data were mapped to the respective genomic assemblies. Where necessary, the region of interest was annotated with Augustus and the gene models were used for FPKM calculations.

Supplemental information

Prediction and analysis of BARS1 and BARS2 3D protein structures

A theoretical 3D model of a plant baruol synthase 1 isoform (NP_193272.1) obtained by the AlphaFold2 algorithm is available (Uniprot ID: O23390). However, an experimental 3D model of any plant OSC is elusive. Thus, we attempted to obtain 3D models of the reference (Col-0) baruol synthase 1 protein (isoforms NP_193272.1, NP_001329547.1) as well as BARS proteins from Cvi-0 encoded by gene duplicates *ATCVI-4G38020* (BARS1) and *ATCVI-4G38110* (BARS2). A comparison of NP_193272.1 models generated with ColabFold software (this study) with that from the UniProt database indicated a very high agreement of their geometrical parameters. Both models superposed with rmsd of ~0.42 Å for 754 Cα atoms, out of overall 759 Cα atoms. Also, the prediction quality for all models we generated with ColabFold was characterized by high pLDDT measures. For the first best predictions, these measures were equal to 91.8 (NP_193272.1), 94.3 (ATCVI-4G38020) and 94.9 (NP_001329547.1 and ATCVI-4G38110). These results indicated that our predictions could be treated with confidence. Therefore, we used 3D protein models obtained with the ColabFold software for further comparative analyses.

Amino acid sequences of Col-0 and Cvi-0 homologs were very similar. Not surprisingly, their structural models superimposed each other very well, with rmsd values from 0.26 Å to 0.42 Å for the Cα atoms (**Supplemental Table S10**). On the other hand, a simple structural comparison of these plant enzymes did not provide any information about their active site. Therefore, we surveyed the Protein Data Bank in search of any OSC experimental structure determined in a complex with its ligand. Using an amino acid sequence of the Col-0 baruol synthase 1 enzyme NP_193272.1, we identified a crystal structure of human OSC (~35% sequence identity) in a complex with lanosterol (ID 1W6K). Superposition of our 3D models of BARS homologs with human OSC revealed their structural similarity, with rmsd values around 1.2 Å for Cα atoms. Moreover, the presence of lanosterol molecule in the active site of the human enzyme allowed us to identify potential substrate-binding cavities in plant homologs (**Supplemental data 1-5**). It is of note that the catalytic aspartate residue D455 present in human cyclase had its counterparts in plant BARS homologs (D493 in Col_BARS1 NP_193272.1, D490 in NP_001329547.1 as well as in ATCVI-4G38020 and ATCVI-4G38110).

Protein sequences of genes predicted with Augustus in de novo genomic assemblies

Ecotype Mir-0

Marneral gene cluster

```
>g1_Mir0 96 aa; Best reference protein match NP_199072.1 AT5G42580 499 aa
MFKPERFLVSSISGDEEKIREQAVKYVTFGGGRRTCPAVKLAHIFMETAIGAMVQCFDWR
IKGEKVYMEEAVSGLSLKMAHPLKCTPVVRFDPFSF
```

Ecotype Mitterberg-2-185

Thalianol gene cluster

```
>g1_Mitterberg 749 aa; Best reference protein match NP_199605.1 AT5G47940 749
aa
MGASNHDNDFNSTTNWKLVDGTLIDAISFESSFTANPESDDGIISAAVDHVTKSPLLLLP
PVPNGEPCEITITFAQEHELRQIYIRSSARVYEVYYTKKRRHDKEYLCTVRCGVAIRDEE
VLQIPLTESADSKPVKDLIERKVTDNGNGRTSEDDWVEVKASDDSLLNNEKQDFYEATAE
INDAEPCTSITVRLLSLQDKRCALVDEVYVFADPVDPSESEKEEATGTGNSSSSSLMAMF
MPALLQLSRGKDVRKERDIQVSDKSNSTDPVAIGNTDQIGVSSPVLVDTVAKQVDAATRV
SGEESKPSISCNNVETIMDQLVKKVSMIETILIRFEDQMLKPINSIDARLQLVEKKLEQL
GNKSFESDLGFRKKIPNQDSLRSDTDKTPDTDESDGLTKNTDVVPDSSSIDNSEDCAVVL
PKNRLDNILSKSVELESENSSISGNEMISAEPEISNEEVGHSFEEKPKYSLSINDALASA
LAGLLSSHSITDGKYSQALVITAPEFSSEDDVEIEQKPGTSAHPDDSQVAAEESENRYSS
SLESSTSSQKEPGITPDDSHGTMYGVFKKLDDSFGGDEEAETVVSVSDNALDEEMVTSST
KADCYTERKNLSYKPTEPDSLIHELESSNVTTAKCKGEPSMDDVLKSVLGFQPTTSSVDF
LTPVLDVKFNLENKDSDSKYFFEVLFTGESKTYLDCKNDVFDDNLVSVEDEEELKGPPTD
TLSSVEMNHYATNEMPIHWNGEISEASLI

>g2_Mitterberg 387 aa; Best reference protein match NP_001078731.1 AT5G47970
387 aa
MTVSEAYSPPLFSIAPMMGWTDNHYRTLARLITKHAWLYTEMLAAETIVYQEDNLDSFLA
FSPDQHPIVLQIGGRNLENLAKATRLANAYAYDEINFNCGCPSPKVSGRGCFGALLMLDP
KFVGEAMSVIAANTNAAVTVKCRIGVDDHDSYNELCDFIHIVSSLSPTKHFIIHSRKALL
SGLSPSDNRRIPPLKYEFFFALLRDFPYLKFTINGGINSVVEADAALRSGAHGVMLGRAV
YYNPWHILGHVDTVIYGSPSSGITRRQVLEKYKVYGESVLGKYGKGRPNLRDIVRPLINL
FHSESGNGQWKRRTDAALLHCTTLQSFLDEVLPAIPDYVLDSSAVKEATGREDLFADVQR
LLPPPYEKESLKALERMPTRPVILDEE

>g3_Mitterberg 223 aa; Best reference protein match NP_199607.1 AT5G47960 223
aa
MSKFQSNFNQKIDYVFKVVLIGDSAVGKSQLLARFSRNEFSIESKATIGVEFQTRTLEID
RKTIKAQIWDTAGQERYRAVTSAYYRGAVGAMLVYDITKRQSFDHVARWLEELRGHADKN
IVIMLIGNKTDLGTLRAVPTEDAKEFAQRENLFFMETSALDSNNVEPSFLTVLTEIYRIV
SKKNLVANEEGESGGDSSLLQGTKIVVAGEETESKGKGCCGTS

>g4_Mitterberg 426 aa; Best reference protein match NP_199606.1 AT5G47950 426
aa
MDTMKVETIGKEIIKPSATTPNDLPTLQLSIMDILMPPVYAVAFLFYTKDDLISQEQTSH
TLKTSLSEILTKFHPLAGRVNGVTIKSTDEGAVFVEARVDNCDLSGFLRSPDTESLKQLL
PVDDEPAPTWPLLVVKATYFQCGGMAIGLCISHRLADAASLSIFLQAWAATARGESDSVA
SPEFCSTKLYPAANEAIKIPGEVVKRTSVTKRFVFVASKIEELRKKVASDVVPRPTRVQS
VTSLIWKCAVTASTDKIREKALFQPANLRTKIPSLLSENQIGNFLFNSLTLDGKAGVDIV
ETVKELQKRAEELSGLVQHEEGSSMTIGSRLFGEIINSKFNFELHDMHSVTSWCKIPLYD
ACFGWGSPVWVAGSVSPDLENVTVLIDSKDGQGIEAWVTLHQDNMLLFEQSTELLAFASP
NPSVLI

>g5_Mitterberg 404 aa; Best reference protein match NP_199609.1 AT5G47980 443
aa
MIFFYNLADLAEKSPDIVSTRLRSSLSQALSRFYPLAGKKEGVSISCNDEGAVFTEARTN
LLLSDFLRNIDINSLKILIPTLAPGESLDSRPLLSVQATFFGSGSGVAVEICVSHCICDA
ASVSTFFRGWAATARGDSNDELSTPQFAEVAIHPPADISIHGSPFNALSEVREKCVTNRF
VFESDKITKLKIVAASKSVPSPTRVEAVMSLIWRCARNASHANLIVPRATMMTQSMDLRL
RIPTNVLSPDAIGNLQGVFFLKRGPGSEIEISEVVAEFRKEKEEFNEMIKENVNGGHTNT
TLGQKIMSGIANYMSELKPNIDTYTMSSWCRKAFYEVDFGWGRPAWVGLGHQDIQDGVMY
VLLVDAKDGEGVEAWVGIPEQDMAAFVCDQELLSYASLNPPVLI
```

>g6_Mitterberg 404 aa; Best reference protein match NP_199609.1 AT5G47980 443 aa
MIFFYNLADLAEKSPDIVSTRLRSSLSQALSRFYPLAGKKEGVSISCNDEGAVFTEARTN
LLLSDFLRNIDINSLKILIPTLAPGESLDSRPLLSVQATFFGSGSGVAVEICVSHCICDA
ASVSTFFRGWAATARGDSNDELSTPQFAEVAIHPPADISIHGSPFNALSEVREKCVTNRF
VFESDKITKLKIVAASKSVPSPTRVEAVMSLIWRCARNASHANLIVPRATMMTQSMDLRL
RIPTNVLSPDAIGNLQGVFFLKRGPGSEIEISEVVAEFRKEKEEFNEMIKENVNGGHTNT
TLGQKIMSGIANYMSELKPNIDTYTMSSWCRKAFYEVDFGWGRPAWVGLGHQDIQDGVMY
VLLVDAKDGEGVEAWVGIPEQDMAAFVCDQELLSYASLNPPVLI

>g7_Mitterberg 511 aa; Best reference protein match NP_199610.1 AT5G47990 511 aa
MASMITVDFENCFIFLLLCLFSRLSYDLFFRKTKDLRAGCALPPSPPSLPIIGHLHLILF
VPIHQSFKNISSKYGPLLHLRFFNFPIVLVSSASTAYEIFKAQDVNVSSRPPPPIEESLI
LGSSSFINTPYGDYSKFMKKFMVQKLLGPQALQRSRNIRADELERFYKTLLDKAMKKQTV
EIRNEAMKLTNNTICKMIMGRSCSEENGEAETVRGLVTESIFLTKKHFLGAMFHKPLKKL
GISLFAKELMNVSNRFDELLEKILVEHEEKLQEHHQTSDMLDMLLEAYGDENAEYKITRD
QIKSLFVDLFSAGTEASANTIQWTMAEIIKNPKICERLREEIDSVVGKTRLVQETDLPNL
PYLQAIVKEGLRLHPPGPVVRTFKETCEIKGFYIPEKTRLFVNVYAIMRDPDFWEDPEEF
KPERFLASSRLGEEDEKREDMLKYIPFGSGRRACPGSHLAYTVVGSVIGMMVQHFDWIIK
GEKINMKEGGTMTLTMAHPLKCTPVPRNLNT

>g8_Mitterberg 471 aa; Best reference protein match NP_001032030.1 AT5G48000 477 aa
MSFVWSAAVWVIAVAAVVISKWLYRWSNPKCNGKLPPGSMGLPIIGETCDFFEPHGLYEI
SPFVKKRMLKYGPLFRTNIFGSNTVVLTEPDIIFEVFRQENKSFVFSYPEAFVKPFGKEN
VFLKHGNIHKHVKQISLQHLGSEALKKKMIGEIDRVTYEHLRSKANQGSFDAKEAVESVI
MAHLTPKIISNLKPETQATLVDNIMALGSEWFQSPLKLTTLISIYKVFIARRDALQVIKD
VFTRRKASREMCGDFLDTMVEEGEKEDVIFNEESAINLIFAILVVAKESTSSVTSLAIKF
LAENHKALAELKREHAAILQNRNGKGAGVSWEEYRHQMTFTNMKGCERCRNQRQVSLKHG
STRSYTIPAGWIVAVIPPAVHFNDAIYENPLEFNPWRWEGKELRSGSKTFMVFGGGVRQC
VGAEFARLQISIFIHHLVTTYDFSLAQESEFIRAPLPYFPKGLPIKISQSL

>g9_Mitterberg 764 aa; Best reference protein match NP_001078733.1 AT5G48010 766 aa
MWRLRTGPKAGEDTHLFTTNNYAGRQIWEFDANAGSPQEIAEVEDARHKFSDNTSRFKTT
ADLLWRMQFLREKKFEQKIPRVIIEDARKIKYEDAKKALKRGLLYFTALQADDGHWPAEN
SGPNFYTPPFLICLYITGHLEKIFTPEHVKELLRHIYNMQNEDGGWGLHVESHSVMFCTV
INYVCLRIVGEEVGHDDQRNGCAKAHKWIMDHGGATYTPLIGKALLSVLGVYDWSGCNPI
PPEFWLLPSSFPVNGGTLWIYLRDTFMGLSYLYGKKFVATPTPLILQLREELYPEPYAKI
NWTQTRNRCGKEDLYYPRSFLQDLFWKSVHMFSESILDRWPLNKLIRQRALQSTMALIHY
HDESTRYITGGCLPKAFHMLACWIEDPKSDYFKKHLARVREYIWIGEDGLKIQSFGSQLW
DTALSLHALLDGIDDHDVDDEIKTTLVKGYDYLKKSQITENPRGDHFKMFRHKTKGGWTF
SDQDQGWPVSDCTAESLECCLFFESMPSELIEKKMDVEKLYDAVDYLLYLQSDNGGIAAW
QPVEGKAWLEWLSPVEFLEDTIVEYVECTGSAIAALTQFNKQFPGYKNVEVKRFITKAAK
YIEDMQTVDGSWYGNWGVCFIYGTFFAVRGLVAAGKTYSNCEAIRKAVRFLLDTQNTEGG
WGESFLSCPSKKYTPLKGNSTNVVQTAQALMVLIMGDQMERDPLPVHRAAQVLINSQLDN
GDFPQQEIMGTFMRTVMLHFPTYRNTFSLWALTHYTHALRRLLP

>g10_Mitterberg 355 aa; Best reference protein match NP_568689.1; AT5G48020 355 aa
MELPVVDLSRYLDFSGDELGSDLLESCRQVSRILKETGALIVKDPRCCAQDNDRFIDMME
NYFEKPDDFKRLQQRPNLHYQVGATPEGVEVPRSLVDEEMQEKFKTMPNEYKPHIPKGPD
HKWRYMWRVGPRPSNTRFKELNSEPVIPEGFPEWEEVMDSWGFKMISAVEVVAEMAAIGF
GLPKDAFTSLMKQGPHLLAPTGSDLNCYNEEGTIFAGYHYDLNFLTIHGRSRFPGLYIWL
RNGEKVAVKVPVGCLLIQAGKQIEWLTAGECIAGMHEVVVTSKTKDAITLAKEQNRSLWR
VSSTLFAHIASDAELKPLGHFAESSLASKYPAIPAGEYVEQELSVINLKGNKGFS

Ecotype Dolna-1-40

Tirucalladienol gene cluster

```
>g1_Dolna 318 aa; Best reference protein match NP_198463.1 AT5G36140 318 aa
MYLTIIFLFISSIIFPLLFFLGKHLSNFRYPNLPPGKIGFPLIGETLSFLSAGRQGHPEK
FVTDRVRHFSSGIFKTHLFGSPFAVVTGASGNKFLFTNENKLVISWWPDSVNKIFPSSTQ
TSSKEEAIKTRMLLMPSMKPEALRRYVGVMDEIAQKHFETEWANQDQLIVFPLTKKFTFS
IACRLFLSMDDLERVRKLEEPFTTVMTGVFSIPIDLPGTRFNRAIKASRLLSKEVSTIIR
QRKEELKAGKVSVEQDILSHMLMNIGETKDEDLADKIIALLIGGHDTTSIVCTFVVNYLA
EFPHIYQRVLEGMQIPLL
```

Ecotype Cvi-0

Arabidiol/baruol gene cluster

```
>g1_Cvi 513 aa; Best reference protein match NP_193270.1 AT4G15350 509 aa;
Augustus predicted gene (chr4:8591612-8593232 complement)
MAAMIFILLCLFTFLCYSLFYKKPKDSRANCDRPPSPPSLPIIGHLHLILSNLAHKSFQR
LSSKYGPLLHLRIFHIPIVLVSSASVAYDIFRAQDVNVSFRSTSTFEECLFFGTSGFFQA
PYGDYWKFMRKLMVTKLLGPQALERSRNIRVEEIDRLYKNLLNKAMKKESVEIGEEASKL
SNNVICTMIMGRSCSEDNGEAERMRSLVSEAMALTKKFFLANIFHKPLKMLGISLFEKEI
MSVSHKFDELLEKILVEHEEKMEEHHQGTDMMDVLLEAYRDENATYKITRNQIKSLIVEL
LIAGTDTSATTTQWIMAELINHPKVFERVREEIDLVVGRSRLIQETDLPNLAYLQAVVKE
ALRLHPPGPLVPRTLQESCEIKGYYIPEKTIVIVNSYAVMRDPYVWEDPEEFKPERFLDI
SSSVQEEEISDKILKFIPFASGRRGCPGTNLAYINVETAIGVMVQCFDWIIKGKEVNMSE
AAGTMVLTLAEPLMCTPVARTLNPLPASLRAYS
```

Ecotype Eri-1

Arabidiol/baruol gene cluster

```
>g1_Eri 763 aa; Best reference protein match NP_001329547.1 AT4G15370 756 aa;
Augustus predicted gene (chr4:8744883-8749727 complement)
MWRLRIGAKAKDNTHLFTTNNYVGRQIWEFDANAGSPEELAEVEEARRNFSNNRSRFKAS
ADLLWRMQFLREKKFEQKIPRVIVEDAEKITYEDAKTALRRGILYFTALQADDGHWPAEN
AGSIFFNAPFVICLYITGHLEKIFTHEHRVELLRYMYNHQNEDGGWGLHVESPSNMFCSV
INYICLRILGVEAGHDDKGSACARARKWILDHGGATYSPLIGKAWLSVLGVYDWSGCKPI
PPEFWFLPSFFPVNGGTLWIYLRDIFMGLSYLYGKNFVATSTPLILQLREEIYPDPYTNI
SWRQARNRCAKEDLYYPQSFLQDLFWKGVHVFSENILNRWPFNNLIRQRALRTTMELVHY
HDEATRYITGGSVPKVFHMLACWVEDPESDYFKKHLARVPDFIWIGEDGLKIQSFGSQVW
DTALSLHVFIDGFDDDVDEEIRSTLLKGYDYLEKSQVTENPPGDYMKMFRHMAKGGWTFS
DQDQGWPVSDCTAESLECCLFFESMSSEFIGKKMDVEKLYDAVDFLLYLQSDNGGITAWQ
PADGKTWLEWLSPVEFIEDAVVEHEYVECTGSAIVALAQFNKQFPGYKKEEVERFITKGV
KYIEDLQMVDGSWYGNWGVCFIYGTFFAVRGLVAAGKCYNNCEAIRRAVRFILDTQNTEG
GWGESYLSCPRKKYIPLIGNKTNVVNTGQALMVLIMGNQMKRDPLPVHRAAKVLINSQMD
NGDFPQQEIMGVFKMNVMLHFPTYRNMFTLWALTHYTKALRGL
```

```
>g2_Eri 513 aa; Best reference protein match NP_193270.1 AT4G15350 509 aa;
Augustus predicted gene (chr4:8792845-8794465 complement)
MAAMIFILLCLFTFLCYSLFYKKPKDSRANCDRPPSPPSLPIIGHLHLILSNLAHKSFQR
LSSKYGPLLHLRIFHIPIVLVSSASVAYDIFRAQDVNVSFRSTSTFEECLFFGTSGFFQA
PYGDYWKFMRKLMVTKLLGPQALERSRNIRVEEIDRLYKNLLNKAMKKESVEIGEEASKL
SNNVICTMIMGRSCSEDNGEAERMRSLVSEAMALTKKFFLANIFHKPLKMLGISLFEKEI
MSVSHKFDELLEKILVEHEEKMEEHHQGTDMMDVLLEAYRDENATYKITRNQIKSLIVEL
```

LIAGTDTSATTTQWIMAELINHPKVFERVREEIDLVVGRSRLIQETDLPNLAYLQAVVKE
ALRLHPPGPLVPRTLQESCEIKGYYIPEKTIVIVNSYAVMRDPYVWEDPEEFKPERFLDI
SSSVQEEEISDKILKFIPFASGRRGCPGTNLAYINVETAIGVMVQCFDWIIKGKEVNMSE
AAGTMVLTLAEPLMCTPVARTLNPLPASLRAYS

Ecotype Ler-0

Arabidiol/baruol gene cluster

>g1_Ler 763 aa; Best reference protein match NP_001329547.1 AT4G15370 756 aa; Augustus predicted gene (chr4:9215522-9220364 complement)
MWRLRIGAKAKDNTHLFTTNNYVGRQIWEFDANAGSPEELAEVEEARRNFSNNRSRFKAS
ADLLWRMQFLREKKFEQKIPRVIVEDAEKITYEDAKTALRRGILYFTALQADDGHWPAEN
AGSIFFNAPFVICLYITGHLEKIFTHEHRVELLRYMYNHQNEDGGWGLHVESPSNMFCSV
INYICLRILGVEAGHDDKGSACARARKWILDHGGATYSPLIGKAWLSVLGVYDWSGCKPI
PPEFWFLPSFFPVNGGTLWIYLRDIFMGLSYLYGKNFVATSTPLILQLREEIYPDPYTNI
SWRQARNRCAKEDLYYPQSFLQDLFWKGVHVFSENILNRWPFNNLIRQRALRTTMELVHY
HDEATRYITGGSVPKVFHMLACWVEDPESDYFKKHLARVPDFIWIGEDGLKIQSFGSQVW
DTALSLHVFIDGFDDDVDEEIRSTLLKGYDYLEKSQVTENPPGDYMKMFRHMAKGGWTFS
DQDQGWPVSDCTAESLECCLFFESMSSEFIGKKMDVEKLYDAVDFLLYLQSDNGGITAWQ
PADGKTWLEWLSPVEFIEDAVVEHEYVECTGSAIVALAQFNKQFPGYKKEEVERFITKGV
KYIEDLQMVDGSWYGNWGVCFIYGTFFAVRGLVAAGKCYNNCEAIRRAVRFILDTQNTEG
GWGESYLSCPRKKYIPLIGNKTNVVNTGQALMVLIMGNQMKRDPLPVHRAAKVLINSQMD
NGDFPQQEIMGVFKMNVMLHFPTYRNMFTLWALTHYTKALRGL

>g2_Ler 513 aa; Best reference protein match NP_193270.1 AT4G15350 509 aa; Augustus predicted gene (chr4:9263484-9265104 complement)
MAAMIFILLCLFTFLCYSLFYKKPKDSRANCDRPPSPPSLPIIGHLHLILSNLAHKSFQR
LSSKYGPLLHLRIFHIPIVLVSSASVAYDIFRAQDVNVSFRSTSTFEECLFFGTSGFFQA
PYGDYWKFMRKLMVTKLLGPQALERSRNIRVEEIDRLYKNLLNKAMKKESVEIGEEASKL
SNNVICTMIMGRSCSEDNGEAERMRSLVSEAMALTKKFFLANIFHKPLKMLGISLFEKEI
MSVSHKFDELLEKILVEHEEKMEEHHQGTDMMDVLLEAYRDENATYKITRNQIKSLIVEL
LIAGTDTSATTTQWIMAELINHPKVFERVREEIDLVVGRSRLIQETDLPNLAYLQAVVKE
ALRLHPPGPLVPRTLQESCEIKGYYIPEKTIVIVNSYAVMRDPYVWEDPEEFKPERFLDI
SSSVQEEEISDKILKFIPFASGRRGCPGTNLAYINVETAIGVMVQCFDWIIKGKEVNMSE
AAGTMVLTLAEPLMCTPVARTLNPLPASLRAYS

Ecotype C24

Arabidiol/baruol gene cluster

>g1_C24 78 aa; Best reference protein match NP_001329547.1 AT4G15370 756 aa; Augustus predicted gene (chr4:9580438-9581743 complement)

MWKLIIGSKAGDDIHLFSTNNYVGRQIWEFDAKAGSPEELAEVEEARQNFTDNRSHFKAS
ADLLWRMQFLREKKFEQKIPRVIIEDAEKITYEDAKTALKRGLLYFTALQADDGHWPAEN
AGSIFFNAPFVICMYITGHLERIFTPEHVRELLRYLYNHQNEDGGWGLHIESPSNMFCTV
INYICLRILGVEAGYDDEGSACARARKWILDHGGATYSPLIGKAWLSVLGVYDWSGCKPI
PPEFWLLPSFLPVNGGLKLEGL

Distribution of accessions with varying CYP705A2-BARS1 copy number status

Map available at:
https://www.google.com/maps/d/edit?mid=1ZaAMX-EDYlbBtKbKdBsS06HMjvc&usp=sharing
Last accessed: September 21, 2022.

Annotations used for evaluation of gene expression in individual accessions

Cdm-0

LR881469.1 MAN gene 10564284 10571454 . - . gene_id "g1_Cdm"; gene_name ""; gene_source "MAN";
LR881469.1 MAN transcript 10564284 10571454 . - . gene_id "g1_Cdm"; transcript_id "g1_Cdm.1"; gene_source "MAN";
LR881469.1 MAN exon 10564284 10564400 . - . gene_id "g1_Cdm"; transcript_id "g1_Cdm.1"; exon_number "1"; gene_name ""; gene_source "MAN";
LR881469.1 MAN CDS 10564284 10564400 . - 0 gene_id "g1_Cdm"; transcript_id "g1_Cdm.1"; cds_number "1"; gene_name ""; gene_source "MAN";
LR881469.1 MAN start_codon 10571452 10571454 . - 0 gene_id "g1_Cdm"; transcript_id "g1_Cdm.1"; gene_name ""; gene_source "MAN";
LR881469.1 MAN exon 10564603 10565089 . - . gene_id "g1_Cdm"; transcript_id "g1_Cdm.1"; exon_number "2"; gene_name ""; gene_source "MAN";
LR881469.1 MAN CDS 10564603 10565089 . - 1 gene_id "g1_Cdm"; transcript_id "g1_Cdm.1"; cds_number "2"; gene_name ""; gene_source "MAN";
LR881469.1 MAN exon 10565181 10565227 . - . gene_id "g1_Cdm"; transcript_id "g1_Cdm.1"; exon_number "3"; gene_name ""; gene_source "MAN";
LR881469.1 MAN CDS 10565181 10565227 . - 0 gene_id "g1_Cdm"; transcript_id "g1_Cdm.1"; cds_number "3"; gene_name ""; gene_source "MAN";
LR881469.1 MAN exon 10565313 10565369 . - . gene_id "g1_Cdm"; transcript_id "g1_Cdm.1"; exon_number "4"; gene_name ""; gene_source "MAN";
LR881469.1 MAN CDS 10565313 10565369 . - 0 gene_id "g1_Cdm"; transcript_id "g1_Cdm.1"; cds_number "4"; gene_name ""; gene_source "MAN";
LR881469.1 MAN exon 10567717 10567815 . - . gene_id "g1_Cdm"; transcript_id "g1_Cdm.1"; exon_number "5"; gene_name ""; gene_source "MAN";
LR881469.1 MAN CDS 10567717 10567815 . - 0 gene_id "g1_Cdm"; transcript_id "g1_Cdm.1"; cds_number "5"; gene_name ""; gene_source "MAN";
LR881469.1 MAN exon 10567896 10568147 . - . gene_id "g1_Cdm"; transcript_id "g1_Cdm.1"; exon_number "6"; gene_name ""; gene_source "MAN";
LR881469.1 MAN CDS 10567896 10568147 . - 0 gene_id "g1_Cdm"; transcript_id "g1_Cdm.1"; cds_number "6"; gene_name ""; gene_source "MAN";
LR881469.1 MAN exon 10568297 10568410 . - . gene_id "g1_Cdm"; transcript_id "g1_Cdm.1"; exon_number "7"; gene_name ""; gene_source "MAN";
LR881469.1 MAN CDS 10568297 10568410 . - 0 gene_id "g1_Cdm"; transcript_id "g1_Cdm.1"; cds_number "7"; gene_name ""; gene_source "MAN";
LR881469.1 MAN exon 10568488 10568630 . - . gene_id "g1_Cdm"; transcript_id "g1_Cdm.1"; exon_number "8"; gene_name ""; gene_source "MAN";
LR881469.1 MAN CDS 10568488 10568630 . - 2 gene_id "g1_Cdm"; transcript_id "g1_Cdm.1"; cds_number "8"; gene_name ""; gene_source "MAN";
LR881469.1 MAN exon 10569276 10569449 . - . gene_id "g1_Cdm"; transcript_id "g1_Cdm.1"; exon_number "9"; gene_name ""; gene_source "MAN";
LR881469.1 MAN CDS 10569276 10569449 . - 2 gene_id "g1_Cdm"; transcript_id "g1_Cdm.1"; cds_number "9"; gene_name ""; gene_source "MAN";
LR881469.1 MAN exon 10570301 10570385 . - . gene_id "g1_Cdm"; transcript_id "g1_Cdm.1"; exon_number "10"; gene_name ""; gene_source "MAN";
LR881469.1 MAN CDS 10570301 10570385 . - 0 gene_id "g1_Cdm"; transcript_id "g1_Cdm.1"; cds_number "10"; gene_name ""; gene_source "MAN";
LR881469.1 MAN exon 10570464 10570667 . - . gene_id "g1_Cdm"; transcript_id "g1_Cdm.1"; exon_number "11"; gene_name ""; gene_source "MAN";
LR881469.1 MAN CDS 10570464 10570667 . - 0 gene_id "g1_Cdm"; transcript_id "g1_Cdm.1"; cds_number "11"; gene_name ""; gene_source "MAN";

LR881469.1 MAN exon 10570761 10570850 . - . gene_id "g1_Cdm"; transcript_id "g1_Cdm.1"; exon_number "12"; gene_name ""; gene_source "MAN";
LR881469.1 MAN CDS 10570761 10570850 . - 0 gene_id "g1_Cdm"; transcript_id "g1_Cdm.1"; cds_number "12"; gene_name ""; gene_source "MAN";
LR881469.1 MAN exon 10570951 10571136 . - . gene_id "g1_Cdm"; transcript_id "g1_Cdm.1"; exon_number "13"; gene_name ""; gene_source "MAN";
LR881469.1 MAN CDS 10570951 10571136 . - 0 gene_id "g1_Cdm"; transcript_id "g1_Cdm.1"; cds_number "13"; gene_name ""; gene_source "MAN";
LR881469.1 MAN exon 10571251 10571454 . - . gene_id "g1_Cdm"; transcript_id "g1_Cdm.1"; exon_number "14"; gene_name ""; gene_source "MAN";
LR881469.1 MAN CDS 10571251 10571454 . - 0 gene_id "g1_Cdm"; transcript_id "g1_Cdm.1"; cds_number "14"; gene_name ""; gene_source "MAN";
LR881469.1 MAN stop_codon 10564284 10564286 . - 0 gene_id "g1_Cdm"; transcript_id "g1_Cdm.1"; gene_name ""; gene_source "MAN";
LR881469.1 MAN gene 10573610 10575227 . + . gene_id "g2_Cdm"; gene_name ""; gene_source "MAN";
LR881469.1 MAN transcript 10573610 10575227 . + . gene_id "g2_Cdm"; transcript_id "g2_Cdm.1"; gene_source "MAN";
LR881469.1 MAN exon 10573610 10574509 . + . gene_id "g2_Cdm"; transcript_id "g2_Cdm.1"; exon_number "1"; gene_name ""; gene_source "MAN";
LR881469.1 MAN CDS 10573610 10574509 . + 0 gene_id "g2_Cdm"; transcript_id "g2_Cdm.1"; cds_number "1"; gene_name ""; gene_source "MAN";
LR881469.1 MAN start_codon 10573610 10573612 . + 0 gene_id "g2_Cdm"; transcript_id "g2_Cdm.1"; gene_name ""; gene_source "MAN";
LR881469.1 MAN exon 10574598 10575227 . + . gene_id "g2_Cdm"; transcript_id "g2_Cdm.1"; exon_number "2"; gene_name ""; gene_source "MAN";
LR881469.1 MAN CDS 10574598 10575227 . + 0 gene_id "g2_Cdm"; transcript_id "g2_Cdm.1"; cds_number "2"; gene_name ""; gene_source "MAN";
LR881469.1 MAN stop_codon 10575225 10575227 . + 0 gene_id "g2_Cdm"; transcript_id "g2_Cdm.1"; gene_name ""; gene_source "MAN";
LR881469.1 MAN gene 10583720 10588582 . - . gene_id "g3_Cdm"; gene_name ""; gene_source "MAN";
LR881469.1 MAN transcript 10583720 10588582 . - . gene_id "g3_Cdm"; transcript_id "g3_Cdm.1"; gene_source "MAN";
LR881469.1 MAN exon 10583720 10583830 . - . gene_id "g3_Cdm"; transcript_id "g3_Cdm.1"; exon_number "1"; gene_name ""; gene_source "MAN";
LR881469.1 MAN CDS 10583720 10583830 . - 0 gene_id "g3_Cdm"; transcript_id "g3_Cdm.1"; cds_number "1"; gene_name ""; gene_source "MAN";
LR881469.1 MAN start_codon 10588580 10588582 . - 0 gene_id "g3_Cdm"; transcript_id "g3_Cdm.1"; gene_name ""; gene_source "MAN";
LR881469.1 MAN exon 10583994 10584480 . - . gene_id "g3_Cdm"; transcript_id "g3_Cdm.1"; exon_number "2"; gene_name ""; gene_source "MAN";
LR881469.1 MAN CDS 10583994 10584480 . - 1 gene_id "g3_Cdm"; transcript_id "g3_Cdm.1"; cds_number "2"; gene_name ""; gene_source "MAN";
LR881469.1 MAN exon 10584581 10584627 . - . gene_id "g3_Cdm"; transcript_id "g3_Cdm.1"; exon_number "3"; gene_name ""; gene_source "MAN";
LR881469.1 MAN CDS 10584581 10584627 . - 0 gene_id "g3_Cdm"; transcript_id "g3_Cdm.1"; cds_number "3"; gene_name ""; gene_source "MAN";
LR881469.1 MAN exon 10584702 10584758 . - . gene_id "g3_Cdm"; transcript_id "g3_Cdm.1"; exon_number "4"; gene_name ""; gene_source "MAN";
LR881469.1 MAN CDS 10584702 10584758 . - 0 gene_id "g3_Cdm"; transcript_id "g3_Cdm.1"; cds_number "4"; gene_name ""; gene_source "MAN";
LR881469.1 MAN exon 10585566 10585664 . - . gene_id "g3_Cdm"; transcript_id "g3_Cdm.1"; exon_number "5"; gene_name ""; gene_source "MAN";
LR881469.1 MAN CDS 10585566 10585664 . - 0 gene_id "g3_Cdm"; transcript_id "g3_Cdm.1"; cds_number "5"; gene_name ""; gene_source "MAN";

LR881469.1 MAN exon 10585760 10586011 . - . gene_id "g3_Cdm"; transcript_id "g3_Cdm.1"; exon_number "6"; gene_name ""; gene_source "MAN";
LR881469.1 MAN CDS 10585760 10586011 . - 0 gene_id "g3_Cdm"; transcript_id "g3_Cdm.1"; cds_number "6"; gene_name ""; gene_source "MAN";
LR881469.1 MAN exon 10586133 10586246 . - . gene_id "g3_Cdm"; transcript_id "g3_Cdm.1"; exon_number "7"; gene_name ""; gene_source "MAN";
LR881469.1 MAN CDS 10586133 10586246 . - 0 gene_id "g3_Cdm"; transcript_id "g3_Cdm.1"; cds_number "7"; gene_name ""; gene_source "MAN";
LR881469.1 MAN exon 10586345 10586469 . - . gene_id "g3_Cdm"; transcript_id "g3_Cdm.1"; exon_number "8"; gene_name ""; gene_source "MAN";
LR881469.1 MAN CDS 10586345 10586469 . - 2 gene_id "g3_Cdm"; transcript_id "g3_Cdm.1"; cds_number "8"; gene_name ""; gene_source "MAN";
LR881469.1 MAN exon 10587041 10587125 . - . gene_id "g3_Cdm"; transcript_id "g3_Cdm.1"; exon_number "9"; gene_name ""; gene_source "MAN";
LR881469.1 MAN CDS 10587041 10587125 . - 0 gene_id "g3_Cdm"; transcript_id "g3_Cdm.1"; cds_number "9"; gene_name ""; gene_source "MAN";
LR881469.1 MAN exon 10587336 10587536 . - . gene_id "g3_Cdm"; transcript_id "g3_Cdm.1"; exon_number "10"; gene_name ""; gene_source "MAN";
LR881469.1 MAN CDS 10587336 10587536 . - 0 gene_id "g3_Cdm"; transcript_id "g3_Cdm.1"; cds_number "10"; gene_name ""; gene_source "MAN";
LR881469.1 MAN exon 10587871 10587960 . - . gene_id "g3_Cdm"; transcript_id "g3_Cdm.1"; exon_number "11"; gene_name ""; gene_source "MAN";
LR881469.1 MAN CDS 10587871 10587960 . - 0 gene_id "g3_Cdm"; transcript_id "g3_Cdm.1"; cds_number "11"; gene_name ""; gene_source "MAN";
LR881469.1 MAN exon 10588095 10588280 . - . gene_id "g3_Cdm"; transcript_id "g3_Cdm.1"; exon_number "12"; gene_name ""; gene_source "MAN";
LR881469.1 MAN CDS 10588095 10588280 . - 0 gene_id "g3_Cdm"; transcript_id "g3_Cdm.1"; cds_number "12"; gene_name ""; gene_source "MAN";
LR881469.1 MAN exon 10588379 10588582 . - . gene_id "g3_Cdm"; transcript_id "g3_Cdm.1"; exon_number "13"; gene_name ""; gene_source "MAN";
LR881469.1 MAN CDS 10588379 10588582 . - 0 gene_id "g3_Cdm"; transcript_id "g3_Cdm.1"; cds_number "13"; gene_name ""; gene_source "MAN";
LR881469.1 MAN stop_codon 10583720 10583722 . - 0 gene_id "g3_Cdm"; transcript_id "g3_Cdm.1"; gene_name ""; gene_source "MAN";
LR881469.1 MAN gene 10630272 10631892 . - . gene_id "g4_Cdm"; gene_name ""; gene_source "MAN";
LR881469.1 MAN transcript 10630272 10631892 . - . gene_id "g4_Cdm"; transcript_id "g4_Cdm.1"; gene_source "MAN";
LR881469.1 MAN exon 10630272 10630919 . - . gene_id "g4_Cdm"; transcript_id "g4_Cdm.1"; exon_number "1"; gene_name ""; gene_source "MAN";
LR881469.1 MAN CDS 10630272 10630919 . - 0 gene_id "g4_Cdm"; transcript_id "g4_Cdm.1"; cds_number "1"; gene_name ""; gene_source "MAN";
LR881469.1 MAN start_codon 10631890 10631892 . - 0 gene_id "g4_Cdm"; transcript_id "g4_Cdm.1"; gene_name ""; gene_source "MAN";
LR881469.1 MAN exon 10630999 10631892 . - . gene_id "g4_Cdm"; transcript_id "g4_Cdm.1"; exon_number "2"; gene_name ""; gene_source "MAN";
LR881469.1 MAN CDS 10630999 10631892 . - 0 gene_id "g4_Cdm"; transcript_id "g4_Cdm.1"; cds_number "2"; gene_name ""; gene_source "MAN";
LR881469.1 MAN stop_codon 10630272 10630274 . - 0 gene_id "g4_Cdm"; transcript_id "g4_Cdm.1"; gene_name ""; gene_source "MAN";
LR881469.1 MAN gene 10634775 10638900 . - . gene_id "g5_Cdm"; gene_name ""; gene_source "MAN";
LR881469.1 MAN transcript 10634775 10638900 . - . gene_id "g5_Cdm"; transcript_id "g5_Cdm.1"; gene_source "MAN";
LR881469.1 MAN exon 10634775 10634891 . - . gene_id "g5_Cdm"; transcript_id "g5_Cdm.1"; exon_number "1"; gene_name ""; gene_source "MAN";

LR881469.1 MAN CDS 10634775 10634891 . - 0 gene_id "g5_Cdm"; transcript_id "g5_Cdm.1"; cds_number "1"; gene_name ""; gene_source "MAN";
LR881469.1 MAN start_codon 10638898 10638900 . - 0 gene_id "g5_Cdm"; transcript_id "g5_Cdm.1"; gene_name ""; gene_source "MAN";
LR881469.1 MAN exon 10635022 10635508 . - . gene_id "g5_Cdm"; transcript_id "g5_Cdm.1"; exon_number "2"; gene_name ""; gene_source "MAN";
LR881469.1 MAN CDS 10635022 10635508 . - 1 gene_id "g5_Cdm"; transcript_id "g5_Cdm.1"; cds_number "2"; gene_name ""; gene_source "MAN";
LR881469.1 MAN exon 10635605 10635651 . - . gene_id "g5_Cdm"; transcript_id "g5_Cdm.1"; exon_number "3"; gene_name ""; gene_source "MAN";
LR881469.1 MAN CDS 10635605 10635651 . - 0 gene_id "g5_Cdm"; transcript_id "g5_Cdm.1"; cds_number "3"; gene_name ""; gene_source "MAN";
LR881469.1 MAN exon 10635727 10635783 . - . gene_id "g5_Cdm"; transcript_id "g5_Cdm.1"; exon_number "4"; gene_name ""; gene_source "MAN";
LR881469.1 MAN CDS 10635727 10635783 . - 0 gene_id "g5_Cdm"; transcript_id "g5_Cdm.1"; cds_number "4"; gene_name ""; gene_source "MAN";
LR881469.1 MAN exon 10636211 10636309 . - . gene_id "g5_Cdm"; transcript_id "g5_Cdm.1"; exon_number "5"; gene_name ""; gene_source "MAN";
LR881469.1 MAN CDS 10636211 10636309 . - 0 gene_id "g5_Cdm"; transcript_id "g5_Cdm.1"; cds_number "5"; gene_name ""; gene_source "MAN";
LR881469.1 MAN exon 10636397 10636648 . - . gene_id "g5_Cdm"; transcript_id "g5_Cdm.1"; exon_number "6"; gene_name ""; gene_source "MAN";
LR881469.1 MAN CDS 10636397 10636648 . - 0 gene_id "g5_Cdm"; transcript_id "g5_Cdm.1"; cds_number "6"; gene_name ""; gene_source "MAN";
LR881469.1 MAN exon 10636725 10636838 . - . gene_id "g5_Cdm"; transcript_id "g5_Cdm.1"; exon_number "7"; gene_name ""; gene_source "MAN";
LR881469.1 MAN CDS 10636725 10636838 . - 0 gene_id "g5_Cdm"; transcript_id "g5_Cdm.1"; cds_number "7"; gene_name ""; gene_source "MAN";
LR881469.1 MAN exon 10636946 10637137 . - . gene_id "g5_Cdm"; transcript_id "g5_Cdm.1"; exon_number "8"; gene_name ""; gene_source "MAN";
LR881469.1 MAN CDS 10636946 10637137 . - 0 gene_id "g5_Cdm"; transcript_id "g5_Cdm.1"; cds_number "8"; gene_name ""; gene_source "MAN";
LR881469.1 MAN exon 10637355 10637521 . - . gene_id "g5_Cdm"; transcript_id "g5_Cdm.1"; exon_number "9"; gene_name ""; gene_source "MAN";
LR881469.1 MAN CDS 10637355 10637521 . - 2 gene_id "g5_Cdm"; transcript_id "g5_Cdm.1"; cds_number "9"; gene_name ""; gene_source "MAN";
LR881469.1 MAN exon 10637618 10637702 . - . gene_id "g5_Cdm"; transcript_id "g5_Cdm.1"; exon_number "10"; gene_name ""; gene_source "MAN";
LR881469.1 MAN CDS 10637618 10637702 . - 0 gene_id "g5_Cdm"; transcript_id "g5_Cdm.1"; cds_number "10"; gene_name ""; gene_source "MAN";
LR881469.1 MAN exon 10637929 10638129 . - . gene_id "g5_Cdm"; transcript_id "g5_Cdm.1"; exon_number "11"; gene_name ""; gene_source "MAN";
LR881469.1 MAN CDS 10637929 10638129 . - 0 gene_id "g5_Cdm"; transcript_id "g5_Cdm.1"; cds_number "11"; gene_name ""; gene_source "MAN";
LR881469.1 MAN exon 10638216 10638305 . - . gene_id "g5_Cdm"; transcript_id "g5_Cdm.1"; exon_number "12"; gene_name ""; gene_source "MAN";
LR881469.1 MAN CDS 10638216 10638305 . - 0 gene_id "g5_Cdm"; transcript_id "g5_Cdm.1"; cds_number "12"; gene_name ""; gene_source "MAN";
LR881469.1 MAN exon 10638407 10638592 . - . gene_id "g5_Cdm"; transcript_id "g5_Cdm.1"; exon_number "13"; gene_name ""; gene_source "MAN";
LR881469.1 MAN CDS 10638407 10638592 . - 0 gene_id "g5_Cdm"; transcript_id "g5_Cdm.1"; cds_number "13"; gene_name ""; gene_source "MAN";
LR881469.1 MAN exon 10638697 10638900 . - . gene_id "g5_Cdm"; transcript_id "g5_Cdm.1"; exon_number "14"; gene_name ""; gene_source "MAN";
LR881469.1 MAN CDS 10638697 10638900 . - 0 gene_id "g5_Cdm"; transcript_id "g5_Cdm.1"; cds_number "14"; gene_name ""; gene_source "MAN";

LR881469.1 MAN stop_codon 10634775 10634777 . - 0
gene_id "g5_Cdm"; transcript_id "g5_Cdm.1"; gene_name ""; gene_source "MAN";

Ty-1

LR797800.1 MAN gene 9989227 9995059 . - . gene_id
"g1_Ty1"; gene_name ""; gene_source "MAN";
LR797800.1 MAN transcript 9989227 9995059 . - . gene_id
"g1_Ty1"; transcript_id "g1_Ty1.1"; gene_source "MAN";
LR797800.1 MAN exon 9989227 9989343 . - . gene_id
"g1_Ty1"; transcript_id "g1_Ty1.1"; exon_number "1"; gene_name ""; gene_source "MAN";
LR797800.1 MAN CDS 9989227 9989343 . - 0 gene_id
"g1_Ty1"; transcript_id "g1_Ty1.1"; cds_number "1"; gene_name ""; gene_source "MAN";
LR797800.1 MAN start_codon 9995057 9995059 . - 0
gene_id "g1_Ty1"; transcript_id "g1_Ty1.1"; gene_name ""; gene_source "MAN";
LR797800.1 MAN exon 9989546 9990032 . - . gene_id
"g1_Ty1"; transcript_id "g1_Ty1.1"; exon_number "2"; gene_name ""; gene_source "MAN";
LR797800.1 MAN CDS 9989546 9990032 . - 1 gene_id
"g1_Ty1"; transcript_id "g1_Ty1.1"; cds_number "2"; gene_name ""; gene_source "MAN";
LR797800.1 MAN exon 9990131 9990177 . - . gene_id
"g1_Ty1"; transcript_id "g1_Ty1.1"; exon_number "3"; gene_name ""; gene_source "MAN";
LR797800.1 MAN CDS 9990131 9990177 . - 0 gene_id
"g1_Ty1"; transcript_id "g1_Ty1.1"; cds_number "3"; gene_name ""; gene_source "MAN";
LR797800.1 MAN exon 9990263 9990319 . - . gene_id
"g1_Ty1"; transcript_id "g1_Ty1.1"; exon_number "4"; gene_name ""; gene_source "MAN";
LR797800.1 MAN CDS 9990263 9990319 . - 0 gene_id
"g1_Ty1"; transcript_id "g1_Ty1.1"; cds_number "4"; gene_name ""; gene_source "MAN";
LR797800.1 MAN exon 9991370 9991468 . - . gene_id
"g1_Ty1"; transcript_id "g1_Ty1.1"; exon_number "5"; gene_name ""; gene_source "MAN";
LR797800.1 MAN CDS 9991370 9991468 . - 0 gene_id
"g1_Ty1"; transcript_id "g1_Ty1.1"; cds_number "5"; gene_name ""; gene_source "MAN";
LR797800.1 MAN exon 9991550 9991801 . - . gene_id
"g1_Ty1"; transcript_id "g1_Ty1.1"; exon_number "6"; gene_name ""; gene_source "MAN";
LR797800.1 MAN CDS 9991550 9991801 . - 0 gene_id
"g1_Ty1"; transcript_id "g1_Ty1.1"; cds_number "6"; gene_name ""; gene_source "MAN";
LR797800.1 MAN exon 9991951 9992064 . - . gene_id
"g1_Ty1"; transcript_id "g1_Ty1.1"; exon_number "7"; gene_name ""; gene_source "MAN";
LR797800.1 MAN CDS 9991951 9992064 . - 0 gene_id
"g1_Ty1"; transcript_id "g1_Ty1.1"; cds_number "7"; gene_name ""; gene_source "MAN";
LR797800.1 MAN exon 9992133 9992275 . - . gene_id
"g1_Ty1"; transcript_id "g1_Ty1.1"; exon_number "8"; gene_name ""; gene_source "MAN";
LR797800.1 MAN CDS 9992133 9992275 . - 2 gene_id
"g1_Ty1"; transcript_id "g1_Ty1.1"; cds_number "8"; gene_name ""; gene_source "MAN";
LR797800.1 MAN exon 9992933 9993106 . - . gene_id
"g1_Ty1"; transcript_id "g1_Ty1.1"; exon_number "9"; gene_name ""; gene_source "MAN";
LR797800.1 MAN CDS 9992933 9993106 . - 2 gene_id
"g1_Ty1"; transcript_id "g1_Ty1.1"; cds_number "9"; gene_name ""; gene_source "MAN";
LR797800.1 MAN exon 9993909 9993993 . - . gene_id
"g1_Ty1"; transcript_id "g1_Ty1.1"; exon_number "10"; gene_name ""; gene_source "MAN";
LR797800.1 MAN CDS 9993909 9993993 . - 0 gene_id
"g1_Ty1"; transcript_id "g1_Ty1.1"; cds_number "10"; gene_name ""; gene_source "MAN";
LR797800.1 MAN exon 9994072 9994275 . - . gene_id
"g1_Ty1"; transcript_id "g1_Ty1.1"; exon_number "11"; gene_name ""; gene_source "MAN";
LR797800.1 MAN CDS 9994072 9994275 . - 0 gene_id
"g1_Ty1"; transcript_id "g1_Ty1.1"; cds_number "11"; gene_name ""; gene_source "MAN";

LR797800.1 MAN exon 9994365 9994454 . - . gene_id
"g1_Ty1"; transcript_id "g1_Ty1.1"; exon_number "12"; gene_name ""; gene_source "MAN";
LR797800.1 MAN CDS 9994365 9994454 . - 0 gene_id
"g1_Ty1"; transcript_id "g1_Ty1.1"; cds_number "12"; gene_name ""; gene_source "MAN";
LR797800.1 MAN exon 9994556 9994741 . - . gene_id
"g1_Ty1"; transcript_id "g1_Ty1.1"; exon_number "13"; gene_name ""; gene_source "MAN";
LR797800.1 MAN CDS 9994556 9994741 . - 0 gene_id
"g1_Ty1"; transcript_id "g1_Ty1.1"; cds_number "13"; gene_name ""; gene_source "MAN";
LR797800.1 MAN exon 9994856 9995059 . - . gene_id
"g1_Ty1"; transcript_id "g1_Ty1.1"; exon_number "14"; gene_name ""; gene_source "MAN";
LR797800.1 MAN CDS 9994856 9995059 . - 0 gene_id
"g1_Ty1"; transcript_id "g1_Ty1.1"; cds_number "14"; gene_name ""; gene_source "MAN";
LR797800.1 MAN stop_codon 9989227 9989229 . - 0
gene_id "g1_Ty1"; transcript_id "g1_Ty1.1"; gene_name ""; gene_source "MAN";
LR797800.1 MAN gene 9997423 9999033 . + . gene_id
"g2_Ty1"; gene_name ""; gene_source "MAN";
LR797800.1 MAN transcript 9997423 9999033 . + . gene_id
"g2_Ty1"; transcript_id "g2_Ty1.1"; gene_source "MAN";
LR797800.1 MAN exon 9997423 9998322 . + . gene_id
"g2_Ty1"; transcript_id "g2_Ty1.1"; exon_number "1"; gene_name ""; gene_source "MAN";
LR797800.1 MAN CDS 9997423 9998322 . + 0 gene_id
"g2_Ty1"; transcript_id "g2_Ty1.1"; cds_number "1"; gene_name ""; gene_source "MAN";
LR797800.1 MAN start_codon 9997423 9997425 . + 0
gene_id "g2_Ty1"; transcript_id "g2_Ty1.1"; gene_name ""; gene_source "MAN";
LR797800.1 MAN exon 9998404 9999033 . + . gene_id
"g2_Ty1"; transcript_id "g2_Ty1.1"; exon_number "2"; gene_name ""; gene_source "MAN";
LR797800.1 MAN CDS 9998404 9999033 . + 0 gene_id
"g2_Ty1"; transcript_id "g2_Ty1.1"; cds_number "2"; gene_name ""; gene_source "MAN";
LR797800.1 MAN stop_codon 9999031 9999033 . + 0
gene_id "g2_Ty1"; transcript_id "g2_Ty1.1"; gene_name ""; gene_source "MAN";
LR797800.1 MAN gene 10008336 10014219 . - . gene_id
"g3_Ty1"; gene_name ""; gene_source "MAN";
LR797800.1 MAN transcript 10008336 10014219 . - . gene_id
"g3_Ty1"; transcript_id "g3_Ty1.1"; gene_source "MAN";
LR797800.1 MAN exon 10008336 10008446 . - . gene_id
"g3_Ty1"; transcript_id "g3_Ty1.1"; exon_number "1"; gene_name ""; gene_source "MAN";
LR797800.1 MAN CDS 10008336 10008446 . - 0 gene_id
"g3_Ty1"; transcript_id "g3_Ty1.1"; cds_number "1"; gene_name ""; gene_source "MAN";
LR797800.1 MAN start_codon 10014217 10014219 . - 0
gene_id "g3_Ty1"; transcript_id "g3_Ty1.1"; gene_name ""; gene_source "MAN";
LR797800.1 MAN exon 10008610 10009096 . - . gene_id
"g3_Ty1"; transcript_id "g3_Ty1.1"; exon_number "2"; gene_name ""; gene_source "MAN";
LR797800.1 MAN CDS 10008610 10009096 . - 1 gene_id
"g3_Ty1"; transcript_id "g3_Ty1.1"; cds_number "2"; gene_name ""; gene_source "MAN";
LR797800.1 MAN exon 10009197 10009243 . - . gene_id
"g3_Ty1"; transcript_id "g3_Ty1.1"; exon_number "3"; gene_name ""; gene_source "MAN";
LR797800.1 MAN CDS 10009197 10009243 . - 0 gene_id
"g3_Ty1"; transcript_id "g3_Ty1.1"; cds_number "3"; gene_name ""; gene_source "MAN";
LR797800.1 MAN exon 10009318 10009374 . - . gene_id
"g3_Ty1"; transcript_id "g3_Ty1.1"; exon_number "4"; gene_name ""; gene_source "MAN";
LR797800.1 MAN CDS 10009318 10009374 . - 0 gene_id
"g3_Ty1"; transcript_id "g3_Ty1.1"; cds_number "4"; gene_name ""; gene_source "MAN";
LR797800.1 MAN exon 10010182 10010280 . - . gene_id
"g3_Ty1"; transcript_id "g3_Ty1.1"; exon_number "5"; gene_name ""; gene_source "MAN";
LR797800.1 MAN CDS 10010182 10010280 . - 0 gene_id
"g3_Ty1"; transcript_id "g3_Ty1.1"; cds_number "5"; gene_name ""; gene_source "MAN";

LR797800.1 MAN exon 10010376 10010627 . - . gene_id "g3_Ty1"; transcript_id "g3_Ty1.1"; exon_number "6"; gene_name ""; gene_source "MAN";
LR797800.1 MAN CDS 10010376 10010627 . - 0 gene_id "g3_Ty1"; transcript_id "g3_Ty1.1"; cds_number "6"; gene_name ""; gene_source "MAN";
LR797800.1 MAN exon 10010749 10010862 . - . gene_id "g3_Ty1"; transcript_id "g3_Ty1.1"; exon_number "7"; gene_name ""; gene_source "MAN";
LR797800.1 MAN CDS 10010749 10010862 . - 0 gene_id "g3_Ty1"; transcript_id "g3_Ty1.1"; cds_number "7"; gene_name ""; gene_source "MAN";
LR797800.1 MAN exon 10010961 10011152 . - . gene_id "g3_Ty1"; transcript_id "g3_Ty1.1"; exon_number "8"; gene_name ""; gene_source "MAN";
LR797800.1 MAN CDS 10010961 10011152 . - 0 gene_id "g3_Ty1"; transcript_id "g3_Ty1.1"; cds_number "8"; gene_name ""; gene_source "MAN";
LR797800.1 MAN exon 10012414 10012580 . - . gene_id "g3_Ty1"; transcript_id "g3_Ty1.1"; exon_number "9"; gene_name ""; gene_source "MAN";
LR797800.1 MAN CDS 10012414 10012580 . - 2 gene_id "g3_Ty1"; transcript_id "g3_Ty1.1"; cds_number "9"; gene_name ""; gene_source "MAN";
LR797800.1 MAN exon 10012690 10012774 . - . gene_id "g3_Ty1"; transcript_id "g3_Ty1.1"; exon_number "10"; gene_name ""; gene_source "MAN";
LR797800.1 MAN CDS 10012690 10012774 . - 0 gene_id "g3_Ty1"; transcript_id "g3_Ty1.1"; cds_number "10"; gene_name ""; gene_source "MAN";
LR797800.1 MAN exon 10013000 10013200 . - . gene_id "g3_Ty1"; transcript_id "g3_Ty1.1"; exon_number "11"; gene_name ""; gene_source "MAN";
LR797800.1 MAN CDS 10013000 10013200 . - 0 gene_id "g3_Ty1"; transcript_id "g3_Ty1.1"; cds_number "11"; gene_name ""; gene_source "MAN";
LR797800.1 MAN exon 10013537 10013626 . - . gene_id "g3_Ty1"; transcript_id "g3_Ty1.1"; exon_number "12"; gene_name ""; gene_source "MAN";
LR797800.1 MAN CDS 10013537 10013626 . - 0 gene_id "g3_Ty1"; transcript_id "g3_Ty1.1"; cds_number "12"; gene_name ""; gene_source "MAN";
LR797800.1 MAN exon 10013732 10013917 . - . gene_id "g3_Ty1"; transcript_id "g3_Ty1.1"; exon_number "13"; gene_name ""; gene_source "MAN";
LR797800.1 MAN CDS 10013732 10013917 . - 0 gene_id "g3_Ty1"; transcript_id "g3_Ty1.1"; cds_number "13"; gene_name ""; gene_source "MAN";
LR797800.1 MAN exon 10014016 10014219 . - . gene_id "g3_Ty1"; transcript_id "g3_Ty1.1"; exon_number "14"; gene_name ""; gene_source "MAN";
LR797800.1 MAN CDS 10014016 10014219 . - 0 gene_id "g3_Ty1"; transcript_id "g3_Ty1.1"; cds_number "14"; gene_name ""; gene_source "MAN";
LR797800.1 MAN stop_codon 10008336 10008338 . - 0 gene_id "g3_Ty1"; transcript_id "g3_Ty1.1"; gene_name ""; gene_source "MAN";
LR797800.1 MAN gene 10055797 10057417 . - . gene_id "g4_Ty1"; gene_name ""; gene_source "MAN";
LR797800.1 MAN transcript 10055797 10057417 . - . gene_id "g4_Ty1"; transcript_id "g4_Ty1.1"; gene_source "MAN";
LR797800.1 MAN exon 10055797 10056444 . - . gene_id "g4_Ty1"; transcript_id "g4_Ty1.1"; exon_number "1"; gene_name ""; gene_source "MAN";
LR797800.1 MAN CDS 10055797 10056444 . - 0 gene_id "g4_Ty1"; transcript_id "g4_Ty1.1"; cds_number "1"; gene_name ""; gene_source "MAN";
LR797800.1 MAN start_codon 10057415 10057417 . - 0 gene_id "g4_Ty1"; transcript_id "g4_Ty1.1"; gene_name ""; gene_source "MAN";
LR797800.1 MAN exon 10056524 10057417 . - . gene_id "g4_Ty1"; transcript_id "g4_Ty1.1"; exon_number "2"; gene_name ""; gene_source "MAN";
LR797800.1 MAN CDS 10056524 10057417 . - 0 gene_id "g4_Ty1"; transcript_id "g4_Ty1.1"; cds_number "2"; gene_name ""; gene_source "MAN";
LR797800.1 MAN stop_codon 10055797 10055799 . - 0 gene_id "g4_Ty1"; transcript_id "g4_Ty1.1"; gene_name ""; gene_source "MAN";
LR797800.1 MAN gene 10060334 10064607 . - . gene_id "g5_Ty1"; gene_name ""; gene_source "MAN";

LR797800.1 MAN transcript 10060334 10064607 . - . gene_id
"g5_Ty1"; transcript_id "g5_Ty1.1"; gene_source "MAN";
LR797800.1 MAN exon 10060334 10060450 . - . gene_id
"g5_Ty1"; transcript_id "g5_Ty1.1"; exon_number "1"; gene_name ""; gene_source "MAN";
LR797800.1 MAN CDS 10060334 10060450 . - 0 gene_id
"g5_Ty1"; transcript_id "g5_Ty1.1"; cds_number "1"; gene_name ""; gene_source "MAN";
LR797800.1 MAN start_codon 10064605 10064607 . - 0
gene_id "g5_Ty1"; transcript_id "g5_Ty1.1"; gene_name ""; gene_source "MAN";
LR797800.1 MAN exon 10060581 10061067 . - . gene_id
"g5_Ty1"; transcript_id "g5_Ty1.1"; exon_number "2"; gene_name ""; gene_source "MAN";
LR797800.1 MAN CDS 10060581 10061067 . - 1 gene_id
"g5_Ty1"; transcript_id "g5_Ty1.1"; cds_number "2"; gene_name ""; gene_source "MAN";
LR797800.1 MAN exon 10061164 10061210 . - . gene_id
"g5_Ty1"; transcript_id "g5_Ty1.1"; exon_number "3"; gene_name ""; gene_source "MAN";
LR797800.1 MAN CDS 10061164 10061210 . - 0 gene_id
"g5_Ty1"; transcript_id "g5_Ty1.1"; cds_number "3"; gene_name ""; gene_source "MAN";
LR797800.1 MAN exon 10061286 10061342 . - . gene_id
"g5_Ty1"; transcript_id "g5_Ty1.1"; exon_number "4"; gene_name ""; gene_source "MAN";
LR797800.1 MAN CDS 10061286 10061342 . - 0 gene_id
"g5_Ty1"; transcript_id "g5_Ty1.1"; cds_number "4"; gene_name ""; gene_source "MAN";
LR797800.1 MAN exon 10061770 10061868 . - . gene_id
"g5_Ty1"; transcript_id "g5_Ty1.1"; exon_number "5"; gene_name ""; gene_source "MAN";
LR797800.1 MAN CDS 10061770 10061868 . - 0 gene_id
"g5_Ty1"; transcript_id "g5_Ty1.1"; cds_number "5"; gene_name ""; gene_source "MAN";
LR797800.1 MAN exon 10061956 10062207 . - . gene_id
"g5_Ty1"; transcript_id "g5_Ty1.1"; exon_number "6"; gene_name ""; gene_source "MAN";
LR797800.1 MAN CDS 10061956 10062207 . - 0 gene_id
"g5_Ty1"; transcript_id "g5_Ty1.1"; cds_number "6"; gene_name ""; gene_source "MAN";
LR797800.1 MAN exon 10062527 10062718 . - . gene_id
"g5_Ty1"; transcript_id "g5_Ty1.1"; exon_number "7"; gene_name ""; gene_source "MAN";
LR797800.1 MAN CDS 10062527 10062718 . - 0 gene_id
"g5_Ty1"; transcript_id "g5_Ty1.1"; cds_number "7"; gene_name ""; gene_source "MAN";
LR797800.1 MAN exon 10062935 10063101 . - . gene_id
"g5_Ty1"; transcript_id "g5_Ty1.1"; exon_number "8"; gene_name ""; gene_source "MAN";
LR797800.1 MAN CDS 10062935 10063101 . - 2 gene_id
"g5_Ty1"; transcript_id "g5_Ty1.1"; cds_number "8"; gene_name ""; gene_source "MAN";
LR797800.1 MAN exon 10063198 10063282 . - . gene_id
"g5_Ty1"; transcript_id "g5_Ty1.1"; exon_number "9"; gene_name ""; gene_source "MAN";
LR797800.1 MAN CDS 10063198 10063282 . - 0 gene_id
"g5_Ty1"; transcript_id "g5_Ty1.1"; cds_number "9"; gene_name ""; gene_source "MAN";
LR797800.1 MAN exon 10063636 10063836 . - . gene_id
"g5_Ty1"; transcript_id "g5_Ty1.1"; exon_number "10"; gene_name ""; gene_source "MAN";
LR797800.1 MAN CDS 10063636 10063836 . - 0 gene_id
"g5_Ty1"; transcript_id "g5_Ty1.1"; cds_number "10"; gene_name ""; gene_source "MAN";
LR797800.1 MAN exon 10063923 10064012 . - . gene_id
"g5_Ty1"; transcript_id "g5_Ty1.1"; exon_number "11"; gene_name ""; gene_source "MAN";
LR797800.1 MAN CDS 10063923 10064012 . - 0 gene_id
"g5_Ty1"; transcript_id "g5_Ty1.1"; cds_number "11"; gene_name ""; gene_source "MAN";
LR797800.1 MAN exon 10064114 10064299 . - . gene_id
"g5_Ty1"; transcript_id "g5_Ty1.1"; exon_number "12"; gene_name ""; gene_source "MAN";
LR797800.1 MAN CDS 10064114 10064299 . - 0 gene_id
"g5_Ty1"; transcript_id "g5_Ty1.1"; cds_number "12"; gene_name ""; gene_source "MAN";
LR797800.1 MAN exon 10064404 10064607 . - . gene_id
"g5_Ty1"; transcript_id "g5_Ty1.1"; exon_number "13"; gene_name ""; gene_source "MAN";
LR797800.1 MAN CDS 10064404 10064607 . - 0 gene_id
"g5_Ty1"; transcript_id "g5_Ty1.1"; cds_number "13"; gene_name ""; gene_source "MAN";

LR797800.1 MAN stop_codon 10060334 10060336 . - 0
gene_id "g5_Ty1"; transcript_id "g5_Ty1.1"; gene_name ""; gene_source "MAN";

Kn-0

LR797810.1 MAN gene 9446623 9452539 . - . gene_id
"g1_Kn0"; gene_name ""; gene_source "MAN";
LR797810.1 MAN transcript 9446623 9452539 . - . gene_id
"g1_Kn0"; transcript_id "g1_Kn0.1"; gene_source "MAN";
LR797810.1 MAN exon 9446623 9446739 . - . gene_id
"g1_Kn0"; transcript_id "g1_Kn0.1"; exon_number "1"; gene_name ""; gene_source "MAN";
LR797810.1 MAN CDS 9446623 9446739 . - 0 gene_id
"g1_Kn0"; transcript_id "g1_Kn0.1"; cds_number "1"; gene_name ""; gene_source "MAN";
LR797810.1 MAN start_codon 9452537 9452539 . - 0
gene_id "g1_Kn0"; transcript_id "g1_Kn0.1"; gene_name ""; gene_source "MAN";
LR797810.1 MAN exon 9446942 9447428 . - . gene_id
"g1_Kn0"; transcript_id "g1_Kn0.1"; exon_number "2"; gene_name ""; gene_source "MAN";
LR797810.1 MAN CDS 9446942 9447428 . - 1 gene_id
"g1_Kn0"; transcript_id "g1_Kn0.1"; cds_number "2"; gene_name ""; gene_source "MAN";
LR797810.1 MAN exon 9447525 9447571 . - . gene_id
"g1_Kn0"; transcript_id "g1_Kn0.1"; exon_number "3"; gene_name ""; gene_source "MAN";
LR797810.1 MAN CDS 9447525 9447571 . - 0 gene_id
"g1_Kn0"; transcript_id "g1_Kn0.1"; cds_number "3"; gene_name ""; gene_source "MAN";
LR797810.1 MAN exon 9447657 9447713 . - . gene_id
"g1_Kn0"; transcript_id "g1_Kn0.1"; exon_number "4"; gene_name ""; gene_source "MAN";
LR797810.1 MAN CDS 9447657 9447713 . - 0 gene_id
"g1_Kn0"; transcript_id "g1_Kn0.1"; cds_number "4"; gene_name ""; gene_source "MAN";
LR797810.1 MAN exon 9448851 9448949 . - . gene_id
"g1_Kn0"; transcript_id "g1_Kn0.1"; exon_number "5"; gene_name ""; gene_source "MAN";
LR797810.1 MAN CDS 9448851 9448949 . - 0 gene_id
"g1_Kn0"; transcript_id "g1_Kn0.1"; cds_number "5"; gene_name ""; gene_source "MAN";
LR797810.1 MAN exon 9449030 9449281 . - . gene_id
"g1_Kn0"; transcript_id "g1_Kn0.1"; exon_number "6"; gene_name ""; gene_source "MAN";
LR797810.1 MAN CDS 9449030 9449281 . - 0 gene_id
"g1_Kn0"; transcript_id "g1_Kn0.1"; cds_number "6"; gene_name ""; gene_source "MAN";
LR797810.1 MAN exon 9449431 9449544 . - . gene_id
"g1_Kn0"; transcript_id "g1_Kn0.1"; exon_number "7"; gene_name ""; gene_source "MAN";
LR797810.1 MAN CDS 9449431 9449544 . - 0 gene_id
"g1_Kn0"; transcript_id "g1_Kn0.1"; cds_number "7"; gene_name ""; gene_source "MAN";
LR797810.1 MAN exon 9449613 9449755 . - . gene_id
"g1_Kn0"; transcript_id "g1_Kn0.1"; exon_number "8"; gene_name ""; gene_source "MAN";
LR797810.1 MAN CDS 9449613 9449755 . - 2 gene_id
"g1_Kn0"; transcript_id "g1_Kn0.1"; cds_number "8"; gene_name ""; gene_source "MAN";
LR797810.1 MAN exon 9450413 9450586 . - . gene_id
"g1_Kn0"; transcript_id "g1_Kn0.1"; exon_number "9"; gene_name ""; gene_source "MAN";
LR797810.1 MAN CDS 9450413 9450586 . - 2 gene_id
"g1_Kn0"; transcript_id "g1_Kn0.1"; cds_number "9"; gene_name ""; gene_source "MAN";
LR797810.1 MAN exon 9451389 9451473 . - . gene_id
"g1_Kn0"; transcript_id "g1_Kn0.1"; exon_number "10"; gene_name ""; gene_source "MAN";
LR797810.1 MAN CDS 9451389 9451473 . - 0 gene_id
"g1_Kn0"; transcript_id "g1_Kn0.1"; cds_number "10"; gene_name ""; gene_source "MAN";
LR797810.1 MAN exon 9451552 9451755 . - . gene_id
"g1_Kn0"; transcript_id "g1_Kn0.1"; exon_number "11"; gene_name ""; gene_source "MAN";
LR797810.1 MAN CDS 9451552 9451755 . - 0 gene_id
"g1_Kn0"; transcript_id "g1_Kn0.1"; cds_number "11"; gene_name ""; gene_source "MAN";

LR797810.1 MAN exon 9451845 9451934 . - . gene_id
"g1_Kn0"; transcript_id "g1_Kn0.1"; exon_number "12"; gene_name ""; gene_source "MAN";
LR797810.1 MAN CDS 9451845 9451934 . - 0 gene_id
"g1_Kn0"; transcript_id "g1_Kn0.1"; cds_number "12"; gene_name ""; gene_source "MAN";
LR797810.1 MAN exon 9452036 9452221 . - . gene_id
"g1_Kn0"; transcript_id "g1_Kn0.1"; exon_number "13"; gene_name ""; gene_source "MAN";
LR797810.1 MAN CDS 9452036 9452221 . - 0 gene_id
"g1_Kn0"; transcript_id "g1_Kn0.1"; cds_number "13"; gene_name ""; gene_source "MAN";
LR797810.1 MAN exon 9452336 9452539 . - . gene_id
"g1_Kn0"; transcript_id "g1_Kn0.1"; exon_number "14"; gene_name ""; gene_source "MAN";
LR797810.1 MAN CDS 9452336 9452539 . - 0 gene_id
"g1_Kn0"; transcript_id "g1_Kn0.1"; cds_number "14"; gene_name ""; gene_source "MAN";
LR797810.1 MAN stop_codon 9446623 9446625 . - 0
gene_id "g1_Kn0"; transcript_id "g1_Kn0.1"; gene_name ""; gene_source "MAN";
LR797810.1 MAN gene 9454903 9456513 . + . gene_id
"g2_Kn0"; gene_name ""; gene_source "MAN";
LR797810.1 MAN transcript 9454903 9456513 . + . gene_id
"g2_Kn0"; transcript_id "g2_Kn0.1"; gene_source "MAN";
LR797810.1 MAN exon 9454903 9455802 . + . gene_id
"g2_Kn0"; transcript_id "g2_Kn0.1"; exon_number "1"; gene_name ""; gene_source "MAN";
LR797810.1 MAN CDS 9454903 9455802 . + 0 gene_id
"g2_Kn0"; transcript_id "g2_Kn0.1"; cds_number "1"; gene_name ""; gene_source "MAN";
LR797810.1 MAN start_codon 9454903 9454905 . + 0
gene_id "g2_Kn0"; transcript_id "g2_Kn0.1"; gene_name ""; gene_source "MAN";
LR797810.1 MAN exon 9455884 9456513 . + . gene_id
"g2_Kn0"; transcript_id "g2_Kn0.1"; exon_number "2"; gene_name ""; gene_source "MAN";
LR797810.1 MAN CDS 9455884 9456513 . + 0 gene_id
"g2_Kn0"; transcript_id "g2_Kn0.1"; cds_number "2"; gene_name ""; gene_source "MAN";
LR797810.1 MAN stop_codon 9456511 9456513 . + 0
gene_id "g2_Kn0"; transcript_id "g2_Kn0.1"; gene_name ""; gene_source "MAN";
LR797810.1 MAN gene 9465744 9471645 . - . gene_id
"g3_Kn0"; gene_name ""; gene_source "MAN";
LR797810.1 MAN transcript 9465744 9471645 . - . gene_id
"g3_Kn0"; transcript_id "g3_Kn0.1"; gene_source "MAN";
LR797810.1 MAN exon 9465744 9465854 . - . gene_id
"g3_Kn0"; transcript_id "g3_Kn0.1"; exon_number "1"; gene_name ""; gene_source "MAN";
LR797810.1 MAN CDS 9465744 9465854 . - 0 gene_id
"g3_Kn0"; transcript_id "g3_Kn0.1"; cds_number "1"; gene_name ""; gene_source "MAN";
LR797810.1 MAN start_codon 9471643 9471645 . - 0
gene_id "g3_Kn0"; transcript_id "g3_Kn0.1"; gene_name ""; gene_source "MAN";
LR797810.1 MAN exon 9466018 9466504 . - . gene_id
"g3_Kn0"; transcript_id "g3_Kn0.1"; exon_number "2"; gene_name ""; gene_source "MAN";
LR797810.1 MAN CDS 9466018 9466504 . - 1 gene_id
"g3_Kn0"; transcript_id "g3_Kn0.1"; cds_number "2"; gene_name ""; gene_source "MAN";
LR797810.1 MAN exon 9466604 9466650 . - . gene_id
"g3_Kn0"; transcript_id "g3_Kn0.1"; exon_number "3"; gene_name ""; gene_source "MAN";
LR797810.1 MAN CDS 9466604 9466650 . - 0 gene_id
"g3_Kn0"; transcript_id "g3_Kn0.1"; cds_number "3"; gene_name ""; gene_source "MAN";
LR797810.1 MAN exon 9466725 9466781 . - . gene_id
"g3_Kn0"; transcript_id "g3_Kn0.1"; exon_number "4"; gene_name ""; gene_source "MAN";
LR797810.1 MAN CDS 9466725 9466781 . - 0 gene_id
"g3_Kn0"; transcript_id "g3_Kn0.1"; cds_number "4"; gene_name ""; gene_source "MAN";
LR797810.1 MAN exon 9467606 9467704 . - . gene_id
"g3_Kn0"; transcript_id "g3_Kn0.1"; exon_number "5"; gene_name ""; gene_source "MAN";
LR797810.1 MAN CDS 9467606 9467704 . - 0 gene_id
"g3_Kn0"; transcript_id "g3_Kn0.1"; cds_number "5"; gene_name ""; gene_source "MAN";

LR797810.1 MAN exon 9467800 9468051 . - . gene_id "g3_Kn0"; transcript_id "g3_Kn0.1"; exon_number "6"; gene_name ""; gene_source "MAN";
LR797810.1 MAN CDS 9467800 9468051 . - 0 gene_id "g3_Kn0"; transcript_id "g3_Kn0.1"; cds_number "6"; gene_name ""; gene_source "MAN";
LR797810.1 MAN exon 9468173 9468286 . - . gene_id "g3_Kn0"; transcript_id "g3_Kn0.1"; exon_number "7"; gene_name ""; gene_source "MAN";
LR797810.1 MAN CDS 9468173 9468286 . - 0 gene_id "g3_Kn0"; transcript_id "g3_Kn0.1"; cds_number "7"; gene_name ""; gene_source "MAN";
LR797810.1 MAN exon 9468385 9468576 . - . gene_id "g3_Kn0"; transcript_id "g3_Kn0.1"; exon_number "8"; gene_name ""; gene_source "MAN";
LR797810.1 MAN CDS 9468385 9468576 . - 0 gene_id "g3_Kn0"; transcript_id "g3_Kn0.1"; cds_number "8"; gene_name ""; gene_source "MAN";
LR797810.1 MAN exon 9469838 9470004 . - . gene_id "g3_Kn0"; transcript_id "g3_Kn0.1"; exon_number "9"; gene_name ""; gene_source "MAN";
LR797810.1 MAN CDS 9469838 9470004 . - 2 gene_id "g3_Kn0"; transcript_id "g3_Kn0.1"; cds_number "9"; gene_name ""; gene_source "MAN";
LR797810.1 MAN exon 9470112 9470196 . - . gene_id "g3_Kn0"; transcript_id "g3_Kn0.1"; exon_number "10"; gene_name ""; gene_source "MAN";
LR797810.1 MAN CDS 9470112 9470196 . - 0 gene_id "g3_Kn0"; transcript_id "g3_Kn0.1"; cds_number "10"; gene_name ""; gene_source "MAN";
LR797810.1 MAN exon 9470426 9470626 . - . gene_id "g3_Kn0"; transcript_id "g3_Kn0.1"; exon_number "11"; gene_name ""; gene_source "MAN";
LR797810.1 MAN CDS 9470426 9470626 . - 0 gene_id "g3_Kn0"; transcript_id "g3_Kn0.1"; cds_number "11"; gene_name ""; gene_source "MAN";
LR797810.1 MAN exon 9470963 9471052 . - . gene_id "g3_Kn0"; transcript_id "g3_Kn0.1"; exon_number "12"; gene_name ""; gene_source "MAN";
LR797810.1 MAN CDS 9470963 9471052 . - 0 gene_id "g3_Kn0"; transcript_id "g3_Kn0.1"; cds_number "12"; gene_name ""; gene_source "MAN";
LR797810.1 MAN exon 9471158 9471343 . - . gene_id "g3_Kn0"; transcript_id "g3_Kn0.1"; exon_number "13"; gene_name ""; gene_source "MAN";
LR797810.1 MAN CDS 9471158 9471343 . - 0 gene_id "g3_Kn0"; transcript_id "g3_Kn0.1"; cds_number "13"; gene_name ""; gene_source "MAN";
LR797810.1 MAN exon 9471442 9471645 . - . gene_id "g3_Kn0"; transcript_id "g3_Kn0.1"; exon_number "14"; gene_name ""; gene_source "MAN";
LR797810.1 MAN CDS 9471442 9471645 . - 0 gene_id "g3_Kn0"; transcript_id "g3_Kn0.1"; cds_number "14"; gene_name ""; gene_source "MAN";
LR797810.1 MAN stop_codon 9465744 9465746 . - 0 gene_id "g3_Kn0"; transcript_id "g3_Kn0.1"; gene_name ""; gene_source "MAN";
